# Design Implications for Explanations: A Case Study on Supporting Reflective Assessment of Potentially Misleading Videos

**DOI:** 10.3389/frai.2021.712072

**Published:** 2021-09-27

**Authors:** Oana Inel, Tomislav Duricic, Harmanpreet Kaur, Elisabeth Lex, Nava Tintarev

**Affiliations:** ^1^ Web Information Systems Group, Department of Software Technology, Delft University of Technology, Delft, Netherlands; ^2^ Social Computing Lab, Institute of Interactive Systems and Data Science, Department of Computer Science and Biomedical Engineering, Graz University of Technology, Graz, Austria; ^3^ Social Computing, Know-Center, Graz, Austria; ^4^ Computer Science and Engineering, School of Information, University of Michigan, Ann Arbor, MI, United States; ^5^ Explainable and Reliable AI, Data Science and Knowledge Engineering, Maastricht University, Maastricht, Netherlands

**Keywords:** reflective assessment, explanations and justifications, reflection triggers, online videos, controversial topics, online video deception

## Abstract

Online videos have become a prevalent means for people to acquire information. Videos, however, are often polarized, misleading, or contain topics on which people have different, contradictory views. In this work, we introduce *natural language explanations* to stimulate more deliberate reasoning about videos and raise users’ awareness of potentially deceiving or biased information. With these explanations, we aim to support users in actively deciding and reflecting on the *usefulness* of the videos. We generate the explanations through an end-to-end pipeline that extracts *reflection triggers* so users receive additional information to the video based on its source, covered topics, communicated emotions, and sentiment. In a between-subjects user study, we examine the effect of showing the explanations for videos on three controversial topics. Besides, we assess the users’ alignment with the video’s message and how strong their belief is about the topic. Our results indicate that respondents’ alignment with the video’s message is critical to evaluate the video’s usefulness. Overall, the explanations were found to be useful and of high quality. While the explanations do not influence the perceived usefulness of the videos compared to only seeing the video, people with an *extreme negative alignment* with a video’s message perceived it as less useful (with or without explanations) and felt more confident in their assessment. We relate our findings to cognitive dissonance since users seem to be less receptive to explanations when the video’s message strongly challenges their beliefs. Given these findings, we provide a set of design implications for explanations grounded in theories on reducing cognitive dissonance in light of raising awareness about online deception.

## 1 Introduction

Online videos constitute the most extensive and rapidly growing portion of the Web content, with over 500 h of video being uploaded every day^1^. This increasing prevalence of online video content has changed the landscape for presenting tutorials, news, and opinions, among others. Recent studies also showed that people are more interested in watching news online (Zubiaga, 2019). In general, videos are a powerful vehicle for conveying both spoken and visual stories, including those that can be highly emotionally charged (Berger and Milkman, 2013), misleading (Garcia et al., 2012), deceiving (Vaccari and Chadwick, 2020), or even unverifiable or stereotypical (Beaudoin, 2009). Since videos can now be shared via YouTube links on several social media platforms such as Facebook, Twitter, Reddit, these emotional effects and conveyed information are likely to be amplified due to their broad reach.

This amplified reach of video content is supported by the call for democratization of information (Burgess and Green, 2018). While deliberative democratization promotes equal and consistent distribution of information across users, it has shown negative consequences on the spread of misleading information (Garcia et al., 2012). Part of the problem is the lack of information literacy and related competencies among users (Association, 2000). Studies have pointed out difficulties users have in critical evaluation and use of online information (Hahnel et al., 2020; Fraillon et al., 2020), as well as in identifying reliable and trustworthy information and sources (Walraven et al., 2008). Extensive research has focused on assessing the credibility of tweets (Bhuiyan et al., 2018; Castillo et al., 2011), news (Chen et al., 2015; Popat et al., 2016), or web blogs (Jo et al., 2019); however, videos are less frequently researched. Research has also shown that trying to influence users’ beliefs might strengthen their position rather than encourage reflection (Lewandowsky et al., 2012; Nguyen et al., 2007). We consider that consumers should actively reason about the online videos they consume, particularly regarding high-stakes or controversial topics, rather than being directly informed about potential deceptions. Nevertheless, *reflective assessment or reflective thinking* is a difficult skill to develop and requires substantial cognitive effort.

One solution is to simply provide additional information (Bhuiyan et al., 2018; Kahneman, 2011) to help people reason actively about the videos they consume online, instead of encouraging them to change their opinion. We consider controversial topics, such as, vaccination, Catalonia independence, and free trade suitable for studying the role of reflective thinking. On such topics, more intuitive, emotion-driven assessments can be made. To the best of our knowledge, previous approaches have not studied how to support reflective assessment for controversial topics. Thus, we distinguish from the work dealing with credibility assessment of online information by focusing on *videos*, and by helping people to actively reflect on the usefulness of the content they watch, instead of providing credibility measures. We do not aim to push a particular evaluation of the video content. Rather, we provide a neutral means for users to judge the usefulness of the video for themselves.

In this paper, we introduce *explanations* (*i.e.*, information which can make something clear by giving a detailed description) to stimulate more deliberate reasoning when assessing the *usefulness of online videos to inform a discussion* on controversial topics and ultimately, to raise user’s awareness regarding potential deceiving or biased information contained in such videos. We survey markers used in previous work on credibility assessment that can be especially informative for controversial topics. Our explanations are grounded in these markers, such as source, sentiment, emotion, and controversiality assessment. We refer to these markers as *reflection triggers*. In the literature, *reflection triggers* are defined as factors that can induce reflection (Verpoorten et al., 2011). We generate the natural language explanations through an end-to-end pipeline, which combines information from: *1)* video subtitles, to cover the key concepts or topics in the video, *2)* video comments, to account for people’s opinion on the video and the topics described in the video, and be able to contrast them with the opinions of the video producer (or people that appear in the video) and *3)* the video channel, to account for source information. Thus, our reflection-driven explanations provide information about the video source (*i.e.*, the YouTube channel that posted the video), the emotions evoked by the users’ comments, the sentiment and emotions evoked by key topics mentioned in the video subtitles and video comments, and their controversiality. Thus, according to the categorization in (Verpoorten et al., 2012) regarding online learning, our users *receive information* through the explanations. In determining *video usefulness* based on this information, people naturally reflect on it, making this usefulness evaluation a neutral proxy for users’ reflection.

We conduct a between-subjects survey (*N* = 217) to understand the impact of these explanations as a means to foster reflective assessment about the *usefulness of a video to inform a discussion*. The experimental setup for the survey tests two conditions, watching a video *without explanations* and *with explanations*. In the latter, participants see the explanations only after watching the entire video. The explanations and the follow-up questions in the survey serve as reflective elements.

Our results indicate that the difference in perceived video usefulness between our conditions (watching videos with or without explanations) is not significant. However, participants with extreme negative alignment with the position of the video are most confident about their assessment of video usefulness when seeing the explanations. A qualitative evaluation of the explanations showed that participants find them to provide sufficient information, be truthful, relevant, and clear. Importantly, each reflection trigger in the explanations was found to be relevant for raising awareness and deciding on the *video usefulness to inform a discussion*, with the channel of the video being most frequently mentioned as informative.

Thus, the key contributions^2^ of the paper are:

1. a *video-agnostic method* to *generate natural language explanations* based on *reflection triggers*; the method combines information from video subtitles, video comments, and video channel;
2. a *user-centered evaluation* of the *effectiveness and quality of the generated explanations to support reflective assessments* of socially-driven online content;
3. an *annotated dataset of 960 videos and their user comments* covering the topics of vaccination, Catalonia independence and free trade; annotations include key topics, controversial topics, sentiments and emotions;
4. a *dataset of 960 reflection-driven natural language explanations* (one per video) focusing on reflection triggers regarding video source, controversiality of depicted topics, and sentiments and emotions evoked by both the video and user comments;

The remainder of the paper is structured as follows. Section 2 introduces related work in the area of reflective assessment for online content. Section 3 summarizes the dataset used in our experiments, while Section 4 describes the pipeline to generate the reflective-driven natural language explanations. Section 5 introduces the user study, and Section 6 analyzes the main results and findings. Section 7 consists of a qualitative analysis of user comments, while Section 8 discusses the main findings and implications of our work. Section 9 presents the limitations of our approach and experiment. Finally, Section 10 summarizes our findings and provides future work.

## 2 Related Work

We first describe previous approaches for supporting reflective assessment, in particular, for online content. Next, we describe previous work on automatically extracted credibility markers and reflect on their usefulness as *reflection triggers*. Finally, we highlight the novelty of our contribution.

### 2.1 Supporting Reflective Assessment

The human cognitive process is often defined in terms of dual-system theories, which split thinking into *intuitive thinking* and *reflective thinking* (Evans, 2008; Kahneman and Frederick, 2002). Intuitive thinking is fast and instinctual. Reflective thinking, in contrast, is slower and more analytical. While more cognitively demanding, it can result in more reliable and careful decisions. Unfortunately, reflective thinking is a difficult skill to teach or nurture and is often missing even among people holding a scientific degree (Shtulman, 2013). Research has also shown that trying to correct incorrect beliefs might strengthen people’s initial beliefs rather than correcting them (Lewandowsky et al., 2012; Nguyen et al., 2007). In particular, such ‘backfiring’ is liable to occur when the argument threatens someone’s identity or falls outside the boundaries of what they consider acceptable, and has been found to be challenging for online fact checking (Kriplean et al., 2014). Nevertheless, research on how to design for reflection is still needed (Baumer et al., 2014).

One way to address the problem is to present information with sufficient support and guidance. Extant research supplies evidence for various active reasoning approaches that support critical thinking. In the classroom, pointing out flawed argumentation techniques proved effective to reduce belief in false information (Cook et al., 2017). Similarly, an intervention combining several aspects, including exposure to a lecture on critical thinking or seeing peers’ arguments, can lead to a statistically significant change in beliefs, in the direction of the position best supported by scientific evidence (Holzer et al., 2018).

Outside the classroom setting, there have also been attempts to design systems that support people in reflective thinking about online content (Bhuiyan et al., 2018; Gupta et al., 2014; Chen et al., 2015). Chen et al. (2015) introduce a methodology to help users detect clickbait that is disguised as online news. Tools have also been developed to help people reflect on the credibility of tweets, *i.e.*, TweetCred (Gupta et al., 2014) and FeedReflect (Bhuiyan et al., 2018). For example, FeedReflect (Bhuiyan et al., 2018) uses visual cues, or nudges, in the form of tooltips, to indicate credibility, as well as questions to encourage users to reflect on tweets credibility. The EVON tool (Hahnel et al., 2020), developed to understand how university students evaluate online information provided by a search engine, proved useful to support users in self-reflection. In the media domain, (Teyssou et al., 2017), present a browser plug-in^3^ to support journalists in verifying user-generated Web videos, through video and channel metadata, comment analysis, external search and Twitter timeline analysis, but no systematic evaluation has yet been published.

To the best of our knowledge, there are not many studies looking at the assessment of videos in computer science. However, in the health domain, videos have been extensively evaluated for credibility (see (Madathil et al., 2015) for a review). These studies consist of manual annotation of fixed criteria and include the markers which we identify in Section 2.2 to use as *reflection triggers* in our explanations. These markers include the source, substantiated or contradictory claims (controversiality), and polarity (sentiment/emotion) and can be automatically extracted.

### 2.2 Assessment With Credibility Markers

We now review markers previously found to be useful for credibility assessment and adapt them for reflective assessment of online videos. The majority of previous approaches in credibility assessment are data-driven, *i.e.*, they are more informed by which features can be automatically derived to support machine learning predictions, and are rarely evaluated in user studies (one notable exception is Jo et al. (2019)). Moreover, to the best of our knowledge, they have not been applied in the context of reflective assessment.

#### 2.2.1 Source of Information

Previous work suggests that the source of information is a valuable credibility signal (Castillo et al., 2011). studied the credibility of information spread on Twitter. They treat credibility assessment as a machine learning problem and evaluate the markers for their predictive power. They find that information about the source is one of the best performing feature to predict the credibility of news events on Twitter (Jo et al., 2019). studied how humans assess web blogs credibility, and also found the source of information to be an important marker. Similarly (Al-Khalifa and Al-Eidan, 2011), use the presence of links to authoritative/reputable news sources and whether the tweet was created by a verified user. Correspondingly, in this study, we extract source-based, *i.e.*, **channel-based** reflection triggers such as the number of video channel subscribers to generate explanations about the source of information.

#### 2.2.2 Sentiment and Emotion

Sentiment information helps users make credibility assessments (Kawai et al., 2008; Zhang et al., 2011). O’Donovan et al. (2012) find that negative tweet sentiment is associated with tweet credibility (Castillo et al., 2011). use sentiment, among others, as a feature to predict tweet credibility (Wanas et al., 2008). find that users associate the presence of emotions to the credibility of discussions in online fora (Giachanou et al., 2019). show that emotional signals in data can help discriminate credible and non-credible information in a fact-checking website. Thus, we also use **sentiment** and **emotion** as reflection triggers. We determine the sentiment and emotion of entities extracted from video subtitles and users’ comments to generate explanations about their sentiment and emotions.

#### 2.2.3 Controversial Topics

We conduct our study specifically on **controversial topics**, *i.e.*, topics on which people have diverse views. Controversy arises as soon as there are sufficiently different or contradictory views about a subject, especially when it is hard or even impossible for one to judge where the truth lies. Controversy is unavoidable, as it occurs for many topics (Rad and Barbosa, 2012). However, if we know that a topic is controversial, a credibility assessment is expected to be difficult to make. Moreover, it may help moderate reactions to strong emotions or sentiment. Previous work has found initial indications that controversiality lexicons contribute to some extent toward detecting controversial tweets (Popescu and Pennacchiotti, 2010). In our work, we adapt the detection method of (Kittur et al., 2007) (Section 4.1.4).

### 2.3 Novelty of the Contribution

Our approach is different from previous approaches in that it does not steer the user in a specific direction (*i.e.*, by either promoting or demoting content). In contrast, previous approaches take a position on the quality/credibility of online content. To the best of our knowledge, our approach is also the first to focus on supporting reflective assessment for videos specifically, rather than tweets or written articles. We approach this with a controlled study, where we evaluate the effectiveness of explanations aimed at supporting reflective assessment for videos. This allows us to support assessments that may be more difficult to make and highly subjective. Finally, while questions are widely used to foster reflective thinking (Baumer et al., 2014), natural language explanations are less researched, to the best of our knowledge.

## 3 Dataset

We selected videos from YouTube on three controversial topics: vaccination, free trade and Catalonia independence. We selected these topics from the list of controversial topics in Wikipedia^4^. The topics vary in terms of how much knowledge people have about them and how emotional they are. We expect that vaccination and Catalonia independence evoke more prominent emotional responses, and that people have limited knowledge about Catalonia independence and free trade. However, our dataset collection methodology can be applied to any topic.

We collected the dataset through the YouTube Data API^5^, by using each of the following search queries: vaccination, Catalonia independence and free trade. We selected videos in English, shorter than 10 min and published before June 21, 2019. The final dataset consists of 960 videos, as shown in Table 1: 285 videos on vaccination, 354 videos on Catalonia independence and 321 videos on free trade. A majority of the videos (64%) are between 1 and 5 min long (inclusive). For each video in our dataset, we extract metadata, channel information, and video comments using the YouTube Data API and the subtitles through the Speech Transcription feature from the Google Cloud Video Intelligence API^6^, the same tool used by YouTube to automatically generate captions.

**TABLE 1:** Overview of the video dataset.

**Topic**	**# Of videos**	**Video duration (s)**			**Videos per channels**				**Comments per video**				
		Min	Max	Avg	#	Min	Max	Avg	#	Min	Max	Avg	Without comments
Vaccination	285	7	597	223.03	232	1	7	1.23	113,408	0	15,833	648	111
Catalonia Indep	354	4	597	194.64	174	1	39	2.03	24,171	0	1,872	89	89
Free Trade	321	3	595	266.92	245	1	9	1.31	13,306	0	675	57	83
All Topics	960	3	597	227.24	611	1	40	1.57	137,951	0	15,712	203	283

*Channels:* The videos were published on 611 unique channels, with the majority of the channels containing one video and at most 40 videos (Ruptly).

*Video Comments:* We extracted all English comments published by 6^*th*^ of August 2019. We excluded all comment replies because we consider they could potentially *1)* generate undesired controversiality and *2)* focus the discussion on misleading topics. We exclude the comment replies because they could potentially target the person that posted the previous comment. We find such comments irrelevant for our purpose of understanding the emotions and sentiments perceived by the community on online videos, as existing literature already shows (Shetty et al., 2020; Kavitha et al., 2020). We removed 12,934 (8.57%) comment replies, and we were left with 137,951 comments. The topic of vaccination generated the most comments compared to the other topics, *e.g.*, one video had 15,833 comments. 283 videos had no user comments.

## 4 Explanation Generation Methodology

In this section, we describe the video-agnostic pipeline developed to generate explanations for helping users reflect on online videos they watch. We use the video subtitles, video comments and video channel to extract and generate the *reflection triggers* which form the explanation. Note that we define explanations as any information, which makes something (*e.g.*, a topic) clear by providing a detailed description. From the textual content of our videos, subtitles and comments, *we extract key topics - represented by key entities*, assess their sentiment (positive, negative, neutral) and emotion (anger, fear, joy, sadness, disgust) and *determine whether they are discussed controversially*.

We focus on key entities mentioned in the video subtitles and comments to account for the main topics discussed in the video and understand how they are perceived in the video itself and users’ comments (through sentiments and emotions). Key entities such as people, locations, events, objects, among others, are known to contextualize information from videos (Gligorov et al., 2011). Furthermore, we focus on the aggregated emotions in video comments, to account for the overall opinion of the users that watched the video, instead of, for example, showing just a sample of polarized comments. On the one hand, we hypothesize that such aggregated views are more informative to foster reflection. On the other hand, we take a privacy-preserving approach, where information provided by end-users (*i.e.*, users that posted comments on the videos) stays in the neutral zone and is not shared. Thus, we provide a solution that discourages the perpetuation of online deception in social networks (Aïmeur et al., 2018). We chose the five emotions, namely anger, fear, joy, sadness and disgust because they are among the basic emotions identified by Ekman (1992). We do not include the surprise emotion because we consider this emotion to have both positive and negative valence, so it might not be indicative enough for users. Thus, we extract the following reflection triggers, motivated in Section 2.2:

• **Video channel** (number of subscribers, related channels, registration date of the channel, and publishing date of the video): the information source is a reflection trigger used extensively in online sources research (Castillo et al., 2011; Jo et al., 2019).
• **Emotions depicted by video comments**: emotion analysis is a prime feature for reflective assessment (Mitra et al., 2017; Stieglitz and Dang-Xuan, 2013; Castillo et al., 2011) that deals with people’s opinions on various topics.
• **Sentiment depicted by key entities extracted from video subtitles and video comments**: topics sentiment is also a prime feature to assess information (Kawai et al., 2008; Zhang et al., 2011).
• **Controversiality of key entities in video**: controversial topics are more prone to generating polarized discussions in online forums, such as video comments (Bail et al., 2018).

**FIGURE 1:**
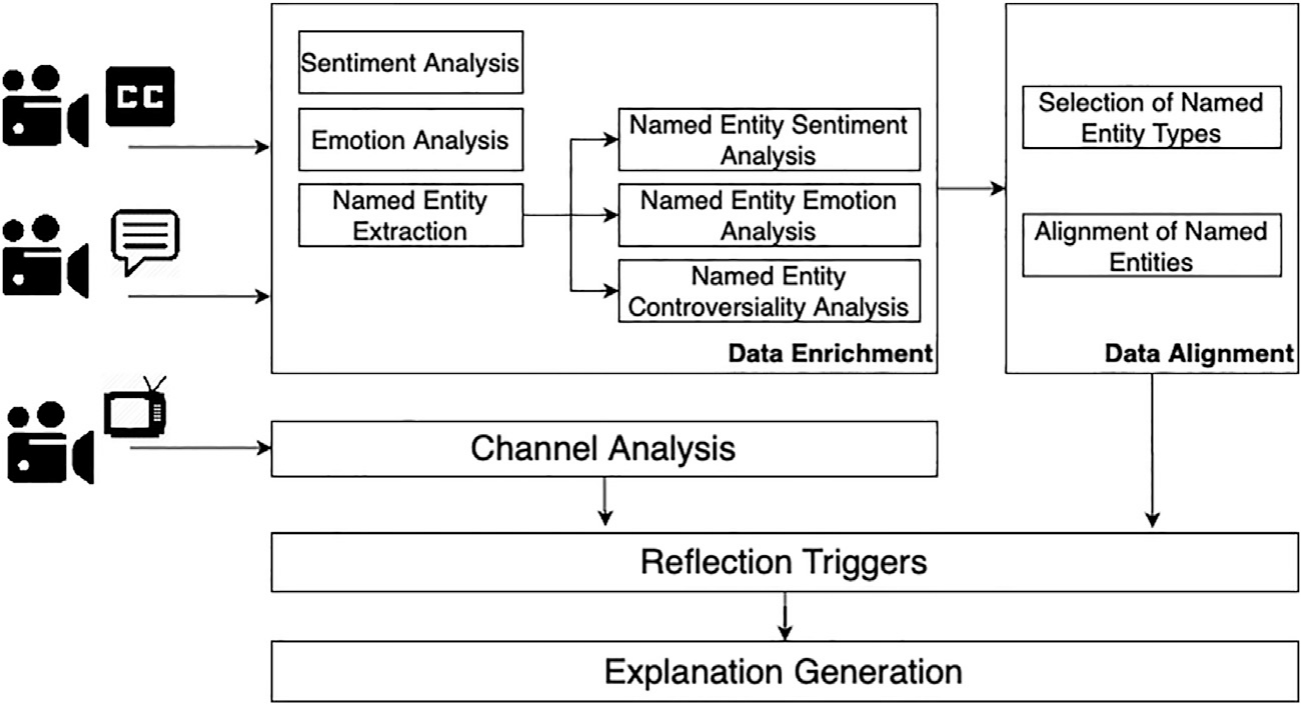
Pipeline for reflective assessment of videos.

Figure 1 depicts the pipeline to extract these triggers. First, we extract key entities from video subtitles and video comments (**data enrichment**) and then we align them (**data alignment**). We next perform **channel analysis** to extract information regarding the source of the video. Finally, using the information from the previous steps, we perform **reflection triggers extraction**, and **explanation generation**.

### 4.1 Data Enrichment

In the data enrichment part, we first extract the sentiment (Section 4.1.1) and the emotion (Section 4.1.2) of each video subtitle and video comment. Then, we extract the named entities from the video subtitles and video comments, their sentiment and emotion scores (Section 4.1.3). Finally, we assign a controversiality score to each named entity (Section 4.1.4). We extract the aforementioned features, using the IBM Watson Natural Language Understanding API^7^ (*Watson NLU*). We chose Watson NLU because it provides all needed functionality for data enrichment, it is easy to use and replicate, through the API, and extensively used in research (Zhu et al., 2014; Canonico and De, 2018; Memeti and Pllana, 2018). A comprehensive comparison^8^ of Watson NLU with other off-the-shelf APIs for text processing found that Watson NLU performed well on entity recognition, and sentiment and emotion analysis, showing, overall, good results quality, comparable with other off-the-shelf tools.

### 4.1.1 Sentiment Analysis

We first extract the sentiment expressed in the video subtitles and each user video comment. The Watson NLU tool returns a sentiment label (positive, negative, neutral) and a sentiment score (between −1.0 and 1.0): neutral sentiment gets a value of 0.0, positive sentiment gets values from (0.0:1.0) and negative sentiment gets values from (−1.0:0.0). Across all video subtitles, the average sentiment score was positive for the free trade topic (0.26), negative for the vaccination topic (−0.24) and close to neutral for the Catalonia independence topic (−0.05). In video comments, however, all average sentiment values were negative, ranging between −0.37 to −0.20, with the topic of vaccination being the most negative and free trade the least negative.

#### 4.1.2 Emotion Analysis

Second, we extract the emotions depicted in the video subtitles and comments. Watson NLU returns a score between 0.0 and 1.0 (the higher the score, the higher the likelihood of the emotion) for each of the following five emotions: *anger*, *disgust*, *fear*, *joy*, and *sadness*. In video subtitles, we observe much higher values for the emotions sadness and joy (between 0.41 and 0.54 for all topics) compared to comments (between 0.24 and 0.30). Overall, video subtitles tend to be more emotionally polarized (*i.e.*, certain emotions stand out on average) than user comments, where the average emotion values are more equally distributed. This could also happen due to the shorter length of user comments compared to video subtitles.

#### 4.1.3 Named Entity Extraction

We use Watson NLU to extract named entities of many types^9^ such as “Person”, “Location”, “Company”, from video subtitles and comments. If available, the tool also returns the DBpedia page of the named entity. We extracted 9,997 and 129,710 named entities from video subtitles and comments.

##### Named Entity Sentiment and Emotion Analysis

We then extract the sentiment and the emotion of each named entity previously identified, similarly as in Sections 4.1.1 and 4.1.2. A named entity has the same sentiment and emotion score for all occurrences in a video subtitle or video comment. In video subtitles, the average sentiment score of all the named entities for the topic vaccination is negative, −0.19. For the topics Catalonia independence and free trade, the average values are very close to neutral; −0.05 and 0.03. In comments, the average sentiment scores of all the named entities is negative for all three topics: −0.20 for vaccination and −0.12 for Catalonia independence and free trade.

In video subtitles, the emotion *sadness* has the highest average score for the topics vaccination (0.21) and Catalonia independence (0.26) and the emotion *joy* has the highest average score for the topic free trade (0.25). The emotions *sadness* (from 0.24 to 0.26) and *joy* (from 0.18 to 0.21) have also the highest average scores in video comments.

##### Pre-Processing

To better align the entities, we first extracted the part-of-speech tags for the named entities previously identified using the NLTK POS tagger^10^. Then, we extracted the lemma of each named entity, using the NLTK WordNet Lemmatizer^11^ and transformed the lemmas to lowercase values, for better aggregation.

#### 4.1.4 Named Entity Controversiality

We extract **controversiality**, which, in our case, expresses whether a named entity appears as controversial on Wikipedia, namely on the page *List of controversial issues* ^12^. A new revision of the page is created when a new topic becomes controversial, or a controversial topic is not controversial anymore. Thus, similarly to Kittur et al. (2007), we check whether we find the Wikipedia page of the named entity in any revision of the aforementioned page. We use the Sparql wrapper^13^ to identify the Wikipedia page from which the DBpedia page of the entity was derived. If the Wikipedia page appears in any revision of the page *List of controversial issues*, the named entity is assigned a controversiality score of 1, and 0 otherwise. We identified 149 named entities (after the alignment in Section 4.2) with a controversiality score of 1.

### 4.2 Data Alignment

We continue with the alignment of the entities identified in video subtitles and video comments, to understand how various key entities are depicted in the two data sources.

#### 4.2.1 Selection of Named Entity Types

To constrain the length of explanations, we only selected five named entity types by looking simultaneously at their frequency in all three topics and their sentiment scores. We first made the selection based on type frequency because we wanted to generate explanations related to concepts that are often mentioned. Second, we made the selection based on the sentiment score because the dataset contains high frequency named entity types (such as “Quantity”) that are always neutral in both video subtitles and video comments. Such entities, however, may not be useful to reflect on the video. This selection resulted in the following categories: “Person”, “Location”, “Organization”, “Company” and “HealthCondition”, with 3,599 named entities in video subtitles and 97,690 in video comments.

#### 4.2.2 Alignment of Named Entities

We distinguish between named entities that: *1)* appear in both video subtitles and video comments, *2)* appear only in video subtitles, and *3)* appear only in video comments. For this study, we focus on the first category because we consider these concepts to be relevant both for the topic (mentioned in the video) and for the users (mentioned in the comments). Moreover, named entities that appear only in the comments might be a potential source of information that cannot be found in the video, thus providing irrelevant information for the video (Shetty et al., 2020; Kavitha et al., 2020). We cannot assess the relevance of such comments, given that they do not refer to concepts from the video. The alignment is done on pre-processed named entities, namely using their lowercase lemma. We found 734 overlapping named entities, for all three topics: vaccination - 115, free trade - 256 and Catalonia Independence - 363. We refer to these as named entities or key entities, for simplicity.

### 4.3 Channel Analysis

Channel information extraction consists of: number of subscribers, channel registration date, and names of related channels.

The number of subscribers was hidden for 26 channels. For the rest of the channels, the maximum number of subscribers was around 15 million (Jimmy Kimmel Live), and the lowest 0 (for 5 channels), with an average (and median) of 417,302 (15,517) subscribers. The oldest channel in our dataset was created on 2^*nd*^ of July 2005, while the newest channel on May 20, 2019. 336 channels had no related channels, while three channels (CBS Evening News, CBS This Morning, and CBS News) had a maximum of 46 related channels. Across all channels, the average number of related channels is 2.72 (median 0), while for the channels with related channels, the average number of related channels is 6 (median 4).

### 4.4 Reflection Triggers

We now define the specific reflection triggers used in the explanations.

• **Video channel**: number of subscribers, names of the related channels, channel registration date, and video publishing date.
• **Controversiality of key entities in video**: we generate a list of controversial key entities, as in Section 4.1.4.
• **Emotions depicted by video comments**:
  1. We compute per topic and emotion the mean score among all comments. We also compute per video and emotion the mean score among all comments. These values indicate the most expressed emotions per topic and video. We consider an emotion to be expressed in a video comment if its score is above the mean value of that emotion, for the topic.
  2. We compute per topic and per emotion, the mean score among all key entities identified. We also compute per video and per emotion the mean score for all the key entities. We consider an emotion to be expressed by a key entity if its score is above the mean value of that emotion, for all the key entities that appear in the topic.
• **Sentiment depicted by key entities extracted from both video subtitles and video comments**: For each key entity, we compute the mean sentiment score in video subtitles and comments.

### 4.5 Explanation Generation

The extracted reflection triggers allow us to generate explanations according to the template in Table 2, column *Explanation Template*. More examples of explanations are provided in the Supplementary Material. The explanations are as neutral in tone as possible but could convey both positive and negative information about the video. In each explanation, the variable between **[square brackets]** is replaced with the indicated value (*e.g* **[date]** - 2015–02–12). Column *Explanation Instantiation* in Table 2 contains an example of a generated explanation. We refer to key entities as topics, for simplicity. Not all the explanation points below are available for all the videos (*e.g.*, for videos without comments, we can not explain the *emotions expressed in video comments*). In this case, no explanation is generated for the given reflection trigger.

**TABLE 2:** Template for generating the reflection-driven explanations and the instantiation of the explanations for Supplementary Video V1 in our experiments.

Explanation template	Explanation instantiation
**Video channel**	**Video channel**
• The *channel* where the video is published has [number] subscribers	• The *channel* where the video is published has 6,487,834 subscribers
• The *channel* where the video is published was created on [date]. The *video* above was posted on [date]	• The *channel* where the video is published was created on 2014–03–04. The *video* above was posted on 2015–02–12
• The *channel* where the video is published is related to the following channel(s): [list of channels]	• The *channel* where the video is published is related to the following channel(s): Ezra Klein Show, Recode, The Verge, Verge Science, Eater, SB Nation, Curbed, Polygon
**Controversial Topics**	**Controversial Topics**
• The *video* is related to the following *topic(s)*: [list of topics]	• The *video* is related to the following *topic(s)*: polio, us, smallpox, Brooklyn, Edward Jenner, Ohio, Rand Paul, Philippines
• The following [number] *topic(s)* mentioned in the video was/were considered *controversial on Wikipedia*: [list of topics]	• The following *2 topic(s)* mentioned in the video was/were considered *controversial on Wikipedia*: us, Philippines
**Emotions in Video Comments**	**Emotions in Video Comments**
• The *comments of the video* express the following *emotion(s)*: [list of emotions]	• The *comments of the video* express the following *emotion(s)*: anger, sadness, fear, disgust
• The *comments of the video* express the following *emotion(s)* towards the *topic(s)* mentioned above:–[list of emotions]: [list of topics]	• The *comments of the video* express the following *emotion(s)* towards the *topic(s)* mentioned above:–anger: us, Edward Jenner–disgust: us, Brooklyn, Rand Paul–fear: polio, us, smallpox, Brooklyn, Philippines–joy: polio, smallpox, Edward Jenner, Ohio–sadness: polio, us, smallpox, Rand Paul, Philippines
**Sentiments in Video Subtitles and Video Comments**	**Sentiments in Video Subtitles and Video Comments**
• The *video subtitles and the video comments* express the following *sentiment(s)* towards the *topic(s)* mentioned above:–[list of topics]: depicted as [sentiment] in the *video subtitles* and as [sentiment] in the *video comments*	• The *video subtitles and the video comments* express the following *sentiment(s)* towards the *topic(s)* mentioned above:–Brooklyn, Rand Paul: depicted as neutral in the *video subtitles* and as negative in the *video comments*.–polio, us, smallpox, Edward Jenner, Ohio, Philippines: depicted as negative in the *video subtitles* and as negative in the *video comments*

## 5 Experiment

We investigate the influence of reflective triggers in natural language explanations on human assessment of online videos on controversial topics. We set up a between-subjects study, which allows us to measure the effect of the explanations on assessing video usefulness. We chose a between-subjects design (instead of within-subjects) to eliminate the risk of knowledge transfer between conditions. Furthermore, this design minimizes unintended reflection: asking participants to watch the video or answer the same questions twice, might contribute toward a reflective assessment.

### 5.1 Materials

We used nine videos in the study, three for each controversial topic (Catalonia independence, free trade, vaccination). We selected videos of 2–3 min because they have the highest coverage in our dataset. Furthermore, we wanted to avoid participant fatigue, ensure all participants put comparable effort into the study, and have a fair payment for their work. Within this duration range, for each topic, we selected all the videos for which all the reflection triggers described in Section 4.5 are available. For each topic, we selected the top three most viewed videos and with the highest number of comments (*i.e.*, above or close to the average number of comments in Table 1). As such, we were impartial in the selection process, ensuring that the videos used in the study *1)* reached a high number of users, *2)* generated extensive discussions, and *3)* the emotions and sentiments expressed in the comments are the collective opinion of many users (*i.e.*, compared to videos that had potentially more views, but few comments).

**Video statistics:** The nine videos have between 7,333 and 793,066 views, and between 135 and 3,917 comments. The videos were posted on eight different channels, namely CNN, Vox, Daily Mail, BBC News, Grandayy, Brexit Party MEPs, RT, and Nuclear Family, between 2013-09-12 and 2019–02-24. The channels have between 31,173 and 7,510,000 subscribers and were created between 2005–10-02 and 2014–07-02. There are two to eight topics mentioned in both the video subtitles and video comments, and one to four controversial topics that refer to locations, people and organizations. The topics identified express the entire range of emotions and sentiments. These statistics show a diverse set of videos. Finally, for each video, we generated the reflection-driven explanations, according to Section 4.5.

### 5.2 Participants

We recruited participants^14^ for our study from the Amazon Mechanical Turk^15^ platform - *master* workers, with at least 95% acceptance rate, and from an English-speaking country (United Kingdom, United States), to ensure high-quality contributions.

### 5.3 Procedure

We asked the participants to imagine the following scenario:

Imagine you are planning to meet a colleague for dinner, and you know (s)he has a strong opinion on a controversial topic that might come up in the conversation. You want to be prepared, so before the dinner, you research the topic on YouTube to learn more.

Each HIT was composed of the assessment of one video, in one of the two conditions: video without explanations (w/o explanations) and video with explanations (w/explanations). Each participant could only participate in one condition, but they could decide how many videos they wanted to assess. The condition w/o explanations lasted for about 3 min, so we paid $0.60 per HIT. The condition w/explanations lasted for about 4 min, so we paid $0.80 per HIT. 35 participants assessed each video. Table 3 shows the variables we measured and the statements the participants rated in our study.

**TABLE 3:** Statements and questions included in the between-subjects user study.

Crt	Variable	Variable type	Statement/Question	Answer space	W/o expl	w/Expl
1	Video Usefulness	Dependent	I find the video useful to inform my opinion, even if I disagree	yes, no, I don’t know	*✓*	*✓*
2	Explanation Usefulness		I find the explanations provided with the video useful to inform my opinion regarding the video	5-point Likert scale		*✓*
3	Confidence		I am confident about my assessment regarding the usefulness of the video		*✓*	*✓*
4	Quantity Enough Information		When making a decision about the usefulness of the video to inform my discussion, the explanations are as informative as they can be			*✓*
5	Quantity Too Much Information		When making a decision about the usefulness of the video to inform my discussion, the explanations give as much information as needed and no more			*✓*
6	Quality		When making a decision about the usefulness of the video to inform my discussion, the explanations are truthful or they do not provide false information, to the best of my knowledge			*✓*
7	Relation		When making a decision about the usefulness of the video to inform my discussion, the explanations are relevant			*✓*
8	Manner		When making a decision about the usefulness of the video to inform my discussion, the explanations are clear, brief, orderly and without obscurity and ambiguity			*✓*
9	Explanation Comp. Usefulness		Which parts of the explanation (if any) helped you think about whether to use the video?	Open-ended		*✓*
10	Alignment Video		The video shares my beliefs about the topic	5-point Likert scale	*✓*	*✓*
11	Belief Strength Topic		I have strong beliefs about the topic of vaccination/Catalonia independence/free trade		*✓*	*✓*
12	Comments	Comment	Comments	Open-ended	*✓*	*✓*

In the condition w/o explanations, participants were first required to watch the video until the end, and then rate the video based on the following criteria: *video usefulness* and *confidence*, *alignment on the video* and *belief strength on the topic*. In the condition w/explanations, the participants were first required to watch the entire video. The explanations were only shown to them after watching the entire video. Then, participants were asked to rate a set of criteria regarding the video and the explanations. To avoid priming and maintain a controlled setup, we do not provide the explanations (simultaneously) with the video. We want the participants to pay attention to the information presented (video and explanation, respectively). Showing them both at once creates a competition for their attention. In addition to the four criteria from the condition w/o explanations, the participants had to rate the *explanation usefulness* and its quality based on the cooperative principles introduced by Grice (1975) (see Section 5.5). Then, participants moved to a second page, which contained the explanations. We asked participants, in a free text field, to tell us which parts of the explanations, if any, helped them reason about the usefulness of the video. According to Verpoorten et al. (2012), such questions can also induce reflection in participants, by encouraging them to verbalize their experience. Participants could also leave comments at the end of the study (see Table 3).

### 5.4 Independent Variables

In our study, we employed two conditions:

1. *without explanations condition* (w/o explanations): assessment of video only;
2. *with explanations condition* (w/explanations): assessment of video and reflective assessment-driven explanations.

### 5.5 Dependent Variables

We measure three variables regarding the reflective assessment of online videos on controversial topics: **video usefulness**, **explanation usefulness**, and **confidence** (rows 1-3 in Table 3). The participants rated **explanations’ usefulness** only in the condition with explanations, using a 5-point Likert scale, from *strongly disagree* to *strongly agree*. The statements regarding **video usefulness** and **confidence** were rated in both conditions–**confidence** on a 5-point Likert scale, from *strongly disagree* to *strongly agree*, and **video usefulness** with the options: *yes*, *no*, and *I don’t know*.

We also measured several dependent variables to evaluate the proposed explanations: **quantity - enough information**, **quantity - too much information**, **quality**, **relation**, **manner**, and **explanation components usefulness** (rows 4–9 in Table 3). Statements 4) to 8) are rated using a 5-point Likert scale from *strongly disagree* to *strongly agree*, while question 9) is open-ended.

We, furthermore, measured two additional variables, in both conditions, using a 5-point Likert scale, from *strongly disagree* to *strongly agree* (entries 10–11 in Table 3): **alignment on the video** and **belief strength on the topic**, for post-hoc analysis. The variable **belief strength on the topic** accounts for participants’ prior knowledge or opinion on the topics.

### 5.6 Hypotheses

We make the following hypotheses for our between-subjects study:

• **H1: (Video Usefulness)** is highest with explanations.
  – **H1a: Video usefulness** is lower for people with extreme assessment of **alignment on the video** (very high and very low), than for more moderate or neutral alignment (Tesser and Conlee, 1975).
  – **H1b: Video usefulness** is lower for people with extreme **topic belief strength** (very high and very low), than for more moderate or neutral topic belief strengths (Nguyen et al., 2007).
• **H2:** The **confidence** is highest with explanations (Kaur et al., 2020).
• **H3:** The ability to make a decision (**Explanation Usefulness**) is highest with explanations (Tintarev, 2007).
• **H4:** The **explanations quality** is high when evaluated using Grice’s Maxims (Grice, 1975):
  – **H4a:** The explanations provide enough information to decide on the usefulness of the video.
  – **H4b:** The explanations provide only the necessary information to decide on the usefulness of the video.
  – **H4c:** The explanations provide truthful information.
  – **H4d:** The explanations are relevant to decide on the usefulness of the video.
  – **H4e:** The explanations are clear, brief, orderly, without obscurity and ambiguity.

### 5.7 Analytical Methods

To analyze the results of the user study, we code participants’ answers as follows: *1)* -1/0/1 for statements evaluated on a 3-point scale (No/I don’t know/Yes); and *2)* with values from 1 to 5 for statements evaluated on a 5-point scale (from strongly disagree to strongly agree).

For hypotheses **H1**, **H2** and **H3** we apply the non-parametric Mann-Whitney U test. For hypotheses **H1a** and **H1b**, measuring the effect of alignment on the video and topic on video usefulness, we apply the Spearman’s rank correlation coefficient, with Bonferroni correction. Further, to test hypotheses **H4a-e** we apply the One-sample *t*-test to see whether the values deviate significantly from the neutral point, 3.0.

## 6 Results of User Study

We analyze the results of our between-subjects user study, using the analytical method from Section 5.7. In total, 217 master workers participated in our study: 68 in the condition w/o explanations (4.6 videos annotated on average per participant) and 149 in the condition w/explanations (2.11 videos annotated on average per participant).

### 6.1 Alignment With the Video and Topic Belief Strength

We investigate whether video usefulness and explanation usefulness are influenced by participants’ belief strength on a topic, and their alignment with the video. The belief strength on the topic implicitly captures participants’ (perceived) prior knowledge on the topic. The alignment on the video captures the agreement with the content of the video. We summarize the descriptive statistics for these variables in Table 4. For both conditions, we observe that: 1) the belief strength on the topic is stronger than the alignment with the videos on vaccination; 2) the lowest belief strength on the topic is recorded on Catalonia independence and the highest for vaccination. For emotional topics about which people have little knowledge (e.g., Catalonia independence), the alignment with the video is higher than the belief strength on the topic. When the topic is less emotionally loaded (e.g., free trade), the alignment with the video is stronger than the belief strength on the topic only in the condition with explanations.

**TABLE 4:** Mean and SD for variables alignment on the video and belief strength on the topic, without and with explanations.

Topic	Video id	Alignment video				Belief strength topic			
		W/o expl		w/Expl		W/o expl		w/Expl	
		Mean	SD	Mean	SD	Mean	SD	Mean	SD
	V1	**4.23**	0.97	**4.20**	0.93	**4.20**	0.96	**4.06**	0.8
V	V2	**4.09**	1.2	**3.91**	0.92	**4.14**	0.91	**3.86**	1.06
	V3	**2.43**	1.4	2.63	1.48	**3.86**	1.06	**3.69**	1.32
All Vaccination		**3.58**	1.45	**3.58**	1.32	**4.07**	0.98	**3.87**	1.08
	CI1	3.06	0.76	2.97	0.66	***2.66*** *	0.97	***2.11*** *	0.93
CI	CI2	**3.29**	0.71	3.14	0.81	**2.20**	1.02	**2.26**	1.27
	CI3	**3.34**	0.87	**3.23**	0.65	**2.37**	0.97	**2.4**	1.09
All Catalonia Indep		**3.23**	0.79	3.11	0.71	**2.41**	1.0	**2.26**	1.1
	FT1	2.94	1.06	3.20	0.76	3.31	0.93	3.06	1.16
FT	FT2	3.09	0.95	3.20	0.80	3.26	1.04	2.89	1.3
	FT3	**3.37**	0.73	**3.63**	0.81	3.17	1.12	3.2	1.08
All free trade		3.13	0.93	**3.34**	0.81	**3.25**	1.03	3.05	1.18
All videos		**3.31**	1.11	**3.35**	1.0	***3.24*** *	1.21	3.06*	1.30

Results are reported by topic (vaccination (V), Catalonia independence (CI), and free trade (FT)), per video (V1‐V3, CI1‐CI3, FT1‐FT3) and for all videos in the study (All Videos). Values range from 1 to 5 for both variables. Statistical significance (*p* < 0.05) is reported in italic* for the Mann Whitney *U* Test and in bold for the One-Sample t‐test.

### 6.2 H1: Video Usefulness

In Table 5, column Video Usefulness, we report on the statistics for the **video usefulness** variable. Video usefulness seems to increase when showing the explanations for the topics of free trade and vaccination, and decrease for the topic of Catalonia independence. However, the differences between the video usefulness in the two conditions, without and with explanations, is not statistically significant (c.f., Mann-Whitney U test for all videos: t = 48,139.0, *p* > 0.05).^16^ Thus, we did not find support for hypothesis **H1**.

**TABLE 5:** Mean and SD for variables video usefulness and confidence, in the conditions without and with explanations.

Topic	Video id	Video Usefulness				Confidence			
		W/o expl		w/Expl		W/o expl		w/Expl	
		Mean	SD.	Mean	SD.	Mean	SD.	Mean	SD.
V	V1	0.94	0.34	0.86	0.49	4.49	0.56	4.46	0.66
	V2	0.69	0.72	0.71	0.67	4.31	0.80	4.17	0.95
	V3	−0.43	0.88	−0.2	0.99	4.23	0.91	4.23	0.91
All vaccination		0.40	0.91	0.46	0.88	4.34	0.77	4.29	0.85
CI	CI1	0.43	0.81	0.49	0.82	4.00	0.69	3.83	0.82
	CI2	0.77	0.60	0.69	0.63	4.09	0.78	3.91	0.98
	CI3	0.77	0.60	0.69	0.63	**4.06**	0.68	**3.71**	0.79
All Catalonia Indep.		0.66	0.69	0.62	0.7	**4.05**	0.71	**3.82**	0.86
FT	FT1	0.43	0.88	0.74	0.56	3.91	0.78	3.77	0.94
	FT2	0.49	0.82	0.54	0.78	3.71	0.99	3.69	0.8
	FT3	0.66	0.73	0.83	0.51	**3.97**	0.89	**4.4**	0.55
All free trade		0.52	0.81	0.70	0.63	3.87	0.89	3.95	0.84
All Videos		0.53	0.81	0.59	0.75	4.09	0.82	4.02	0.87

Results are reported by topic (vaccination (V), Catalonia independence (CI), and free trade (FT)), by individual videos (V1-V3, CI1-CI3, FT1-FT3) and for all videos in the study (All Videos). Values range from −1 to 1 for video usefulness, and from one to 5 for the other variables. Statistical significance (*p* < 0.05) using the Mann Whitney *U* Test is reported in bold.

#### 6.2.1 H1a: Extreme Alignment With the Video Affects Video Usefulness

We hypothesized that **video usefulness** is lower for people with extreme assessment of **video alignment** (very high and very low), than for more neutral alignment.

To represent extreme alignment, we recode participants’ answers for *video alignment* as follows: *strongly disagree* and *strongly agree* with 1 (*extreme*), and the others with -1 (*neutral*). We observe that people found the video slightly more useful in the condition w/explanations, for both people with extreme (w/o explanations - mean = 0.55, SD = 0.83, w/explanations - mean = 0.60, SD = 0.79) and more neutral alignments (w/o explanations - mean = 0.52, SD = 0.81, w/explanations - mean = 0.59, SD = 0.74). We did not find any correlation between video usefulness and extreme and neutral alignments with the video (Spearman’s rank correlation with Bonferroni correction). Thus, we do not find support for hypothesis **H1a**.

In a post-hoc analysis, we investigated whether there is a difference in extreme positive and extreme negative alignment with the video, regarding video usefulness. We recoded participants’ answers for *video alignment* as follows: strongly disagree as -1 to reflect extreme negative alignment, strongly agree as 1 to reflect extreme positive alignment, and the other responses as 0 (neutral). People with extreme negative alignment (w/o explanations: m = −0.72, SD = 0.67, w/explanations: m = −0.29, SD = 0.99) find the video much less useful in both conditions, but especially in the condition w/o explanations. People with extreme positive alignment with the video find the video much more useful in both conditions (w/o explanations: m = 0.95, SD = 0.29, w/explanations: m = 0.87, SD = 0.45). Participants with neutral alignment find the video slightly more useful in the condition w/explanations (w/o explanations: m = 0.52, SD = 0.81, w/explanations: m = 0.59, SD = 0.74). The Spearman’s rank correlation test (Bonferroni corrected) showed a moderate positive correlation between the participants’ alignment on the video (extreme negative, neutral, extreme positive) and the video usefulness (w/o explanations r = 0.37, *p* ≪0.05, w/explanations: r = 0.23, *p* ≪0.05).

#### 6.2.2 H1b: Extreme Belief Strength on the Topic Affects Video Usefulness

**Video usefulness** is lower for people with extreme **belief strength on the topic** (very high and very low), than for more neutral evaluations.

We recode participants’ answers for *topic belief strength*: *strongly disagree* and *strongly agree* as 1 to account for extreme belief strength, while the others as -1 to account for neutral belief strength. The differences in video usefulness are slightly larger for people with extreme belief strength on the topic in the two conditions (w/o explanations - m = 0.49, SD = 0.85; w/explanations - m = 0.66, SD = 0.71), but very similar for more neutral participants (w/o explanations - m = 0.54, SD = 0.79; w/explanations - m = 0.57, SD = 0.77). The Spearman’s rank correlation test (Bonferroni corrected), confirmed, however, that video usefulness is not correlated with the strength of the topic belief, and we do not find support for **H1b**.

For the post-hoc analysis, performed as in H1a, the Spearman’s rank correlation test (Bonferroni corrected), confirmed there is no correlation between the belief strength on the topic (extreme negative, neutral, extreme positive) and the video usefulness, in the two conditions.

### 6.3 H2: Confidence Is Higher With Explanations

In column Confidence in Table 5 we show the statistics for the confidence variable. In general, participants’ confidence seems slightly lower when seeing the explanations. The difference, however, is only statistically significant for video CI3 (c.f., Mann-Whitney U test t = 486.500, *p* < 0.05) and for the topic Catalonia independence (t = 4,848.000, *p* < 0.05)–confidence was lower with explanations; and for video FT3 (t = 458.500, *p* < 0.05)–confidence was higher with explanations. We further discuss these cases in the qualitative analysis in Section 7. Thus, we do not find support for **H2**.

We also analyzed participants’ confidence and their alignment on the video. Participants with extreme positive alignment with the video show slightly lower confidence when seeing explanations (w/o explanations - mean = 4.7, SD = 0.5, w/explanations - mean = 4.54, SD = 0.8). Participants with extreme negative alignment with the video, *i.e.*, who oppose the video, are more confident when seeing explanations (w/o explanations - mean = 4.28, SD = 1.13, w/explanations - mean = 4.71, SD = 0.61). Finally, participants who are more neutral toward the video show slightly lower confidence when seeing explanations (w/o explanations - mean = 3.85, SD = 0.8, w/explanations - mean = 3.8, SD = 0.89).

### 6.4 H3: Explanation Usefulness

Table 6 shows the statistics for the explanation usefulness variable. Overall, participants find the explanations useful to decide on video usefulness. For the majority of the videos (except V3 and CI1), all topics and all videos in the dataset, we find support c. f. One-Sample *t*-Test to conclude that explanations are considered useful (their mean score is statistically significantly higher than the neutral value of 3). We further discuss the two video exceptions in Section 7.

**TABLE 6:** Mean and SD for variables explanations usefulness, explanations quantity 1 - enough information, explanations quantity 2 - too much information, explanations quality, explanations relation and explanations manner in the condition w/explanations.

Topic	Video id	Explanations usefulness		Explanations quantity 1		Explanations quantity 2		Explanations quality		Explanations relation		Explanations manner	
		Mean	SD	Mean	SD	Mean	SD	Mean	SD	Mean	SD	Mean	SD
	V1	**4.31**	0.8	**3.80**	0.96	**3.77**	0.88	**4.00**	0.91	**4.11**	0.72	**4.03**	1.10
V	V2	**3.49**	1.29	**3.43**	1.20	3.40	1.17	**3.97**	0.98	**4.00**	1.16	**3.77**	1.24
	V3	2.97	1.50	2.77	1.24	2.74	1.20	3.09	1.27	3.29	1.07	2.71	1.18
All Vaccination		**3.59**	1.34	**3.33**	1.21	**3.30**	1.16	**3.69**	1.14	**3.80**	1.06	**3.50**	1.29
	CI1	3.29	1.13	3.14	1.03	2.86	1.17	**3.69**	0.93	**3.86**	0.94	3.37	1.11
CI	CI2	**3.80**	0.87	**3.37**	1.06	3.14	1.06	**3.80**	0.58	**4.00**	0.59	**3.60**	0.88
	CI3	**3.86**	0.91	**3.51**	0.98	3.34	1.03	**3.80**	0.76	**3.71**	0.83	**3.86**	0.91
All Catalonia Indep.		**3.65**	1.00	**3.34**	1.03	3.11	1.09	**3.76**	0.77	**3.86**	0.80	**3.61**	0.99
	FT1	**3.63**	0.94	3.31	1.05	3.14	1.06	**3.74**	0.78	**3.69**	0.87	**3.43**	1.07
FT	FT2	**3.66**	0.87	3.26	1.12	3.11	1.11	**3.60**	0.98	**3.77**	0.97	3.29	1.10
	FT3	**4.03**	0.95	**3.69**	1.21	**3.6**	0.98	**4.06**	0.91	**4.14**	0.81	**4.06**	1.00
All free trade		**3.77**	0.93	**3.42**	1.13	**3.29**	1.06	**3.80**	0.9	**3.87**	0.90	**3.59**	1.10
All videos		**3.67**	1.11	**3.37**	1.12	**3.23**	1.11	**3.75**	0.95	**3.84**	0.92	**3.57**	1.13

Results are reported by topic (vaccination (V), Catalonia independence (CI), and free trade (FT)), per video (V1-V3, CI1-CI3, FT1-FT3) and for all videos in the study (All Videos). Values range from 1 to 5 for all variables. Statistical significance (*p* < 0.05) is reported in **bold** c.f. One-Sample *t*-Test.

### 6.5 H4: Explanations Quality Is High

In Table 6 we report on the statistics for the variables **explanations quantity - enough information** (quantity 1), **explanations quantity - too much information** (quantity 2), **explanations quality**, **explanations relation** and **explanations manner**, for all videos, per topic and per video, as measured in the condition w/explanation. The explanation scores of all variables are significantly higher than the neutral value (3.0), for all videos in our user study and each topic, c. f. One-Sample *t*-Test. Following, we look in detail into each variable, per video and hypothesis. Videos for which we do not find support are addressed in the qualitative analysis in Section 7.

#### 6.5.1 H4a: (Explanation Quantity - Enough Information) the Explanations Provide Enough Information to Decide on the Usefulness of the Video

For the majority of the videos in our study, 5 out of 9, we find that enough information is provided in the explanations to decide on the usefulness of the video, thus finding partial support for hypothesis **H4a**.

#### 6.5.2 H4b: (Explanation Quantity - Not Too Much Information) the Explanations Provide Only the Necessary Information to Decide on the Usefulness of the Video

For the majority of the videos (7 out of 9), participants consider the provided explanations neutral (i.e., containing too much information). Thus, we do not find sufficient support for hypothesis **H4b**.

#### 6.5.3 H4c: (Explanation Quality) the Explanations Provide Truthful Information, *i.e.*, They do Not Provide False Information

All the explanations that we generated scored above the neutral value on quality, but for video V3 the difference between the neutral value and the mean quality score of the explanations is not statistically significant. However, we find sufficient support for our hypothesis **H4c**, which states that the explanations are truthful.

#### 6.5.4 H4d: (Explanation Relation) the Explanations Are Relevant to Decide on the Usefulness of the Video

All explanations score above 3.0, in terms of relation. There is also a significant difference in the mean explanation relation value and the neutral value. Thus, we find evidence to support hypothesis **H4d** and conclude that the generated explanations are relevant to decide on video usefulness.

#### 6.5.5 H4e: The Explanations Are Clear, Brief, Orderly, and Without Obscurity and Ambiguity

For all videos, except V3, the explanations generated score on average above the neutral value of 3.0 on explanation manner. Moreover, the difference between these two values is statistically significant for most videos, except for Supplementary Video V3, CI1 and FT2. These differences seem to directly correlate with explanation quantity. Thus, we conclude that we find partial support for hypothesis **H4e**.

### 6.6 Summary

In summary, we found the following:

• **H1**: We did not see differences in *video usefulness* across the two conditions.
  – **H1a**: We found a moderate positive correlation between participants’ *alignment with the video* (high, medium, low) and video usefulness, in both study conditions.
  – **H1b**: The *belief strength on the topic* is not correlated with video usefulness.
• **H2**: Participants’ *confidence* is not statistically significant different between the two conditions.
• **H3**: For the majority of the videos, the participants find the *explanations useful* to decide on video usefulness.
• **H4a-e**: We found sufficient support that the explanations contain *enough information*, are *truthful*, *relevant*, *clear, brief and without ambiguity.* However, we did not find sufficient support to conclude that the explanations do not contain *too much information.*

## 7 Analysis of Comments

In this section, we perform a qualitative analysis of the comments submitted by the participants in the user study. We first analyze the comments regarding explanations and their usefulness submitted in the condition with explanations (entry 9 Table 3). Then, we analyze the general comments submitted in both study conditions (entry 12 Table 3). We used an open-coding approach to extract the main themes (Braun and Clarke, 2006) that appear in these comments.^17^ Two authors of the paper worked together to identify and discuss themes.

### 7.1 Detailed Analysis of Explanatory Reflection Triggers

We received 305 answers regarding explanations, and we extracted the following codes: not relevant - information not relevant for explanations (166 comments), not useful^18^ - explanations are not useful (44 comments), useful - explanations are useful (95 comments). The comments that were marked as useful, were then coded with the reflection trigger(s) they mention: channel, topics, and sentiment and emotions. In Table 7, we show such comment excerpts that we further refer to, as comment #id, in the remainder of the section.

**TABLE 7:** Example of comments given by participants in the user study, when asked the question: *“Which parts of the explanations (if any) helped you think about whether to use the video?”*.

Theme	Number of comments	Comment id	Comment excerpts
Channel	59	#1	*The part in the beginning where is said VOX made me 100% sure I will never use any information in the video*
		#2	*The fact that the video is from a long running channel and a generally reliable news source is the most important*
		#3	*Number of subscribers, video came from a media news source*
		#4	*The channels that the channel that published this video are related to*
		#5	*The time the video was posted…*
		#6	*It doesn’t have many subscribers on the channel*
Topics	26	#7	*I would say the reference of the topic such as where this event was located and Wikipedia were very helpful*
		#8	*The fact that the topic is considered controversial*
		#9	*The topics … accurately depicted the content of the video*
		#10	*While the country (Germany) was brought up for reference, as the meeting was recorded there, it had little to do with the main conversation. Otherwise Good descriptions*
		#11	*the explanation is a little vague and in some parts not correct as in Dr Phil who i only heard mentioned once*
Emotion & Sentiment	34	#12	*…the emotion … felt correct as public opinion was rather split on this issue*
		#13	*The fact that the comments and the subtitles have opposite connotations in relation to Catalonia. It makes me think one or the other is biased in some way*
		#14	*The video subtitles and comments on the video were the most helpful in deciding*
		#15	*The sentiment in the comments seems to be one-sided, and I felt the video only covered one side of the issue, so I felt the video wasn’t that useful*
		#16	*I never put a lot of credence into comments on something like that because it is usually a sea of emotion…*
		#17	*I don’t understand how Catalonia is shown as “negative” in the subtitles. To me it was depicted as positive*
Not Useful	44	#18	*The video was clear enough that I didn’t need the explanation. It also already came from a good source (Vox)*
		#19	*I used the video only as my guide*
		#20	*The explanation did not help me … seems contradictory, particularly looking at the emotions depicted in the comments*
		#21	*I do not think the explanations were as needed … some of the explanations were a little light on actually explaining*
		#22	*I don’t think the explanations showed that this is a fake video*

#### 7.1.1 Reflection Triggers

Among the 95 comments that mentioned the usefulness of the explanations, 59 referred to the channel, 26 to the topics identified and 34 to the sentiments and emotions evoked by the video subtitles and video comments. In addition, four comments mentioned the usefulness of the explanations, as a whole. In general, the reflection trigger referring to the video channel is the most prominently mentioned as being relevant. Participants also paid attention to the factuality and correctness of these reflection triggers, and they agree with the values provided for topics, emotions and sentiments.

*Source.* The channel (source) of the video appears in most comments, suggesting that the video source is a powerful reflection trigger to decide on the usefulness of a video. Furthermore, all components regarding the source are mentioned in the comments: *1)* channel names (comment #1), *2)* channel longevity (comment #2), *3)* number of subscribers of the channel (comments #3, #6), *4)* related channels (comment #4), and *5)* video publishing date (comment #5).

*Topics.* Participants appreciate the connection with the Wikipedia pages (comment #7) and the mention of controversial topics in the video (comment #8). Furthermore, participants appreciate the objective, factual description of the topics and their correctness (comment #9). Comments (#10, #11) also suggest that some of the topics identified are not very relevant, providing new directions for future work (*i.e.*, emphasize topics’ relevance).

*Emotions and sentiment.* Emotion and sentiment triggers seem to generate opposing views regarding video usefulness. Participants acknowledge that these reflection triggers are in general useful/correct (comments #12, #14), and that they make them reflect on the video usefulness (comments #13, #15). However, they also expressed concerns about the use of comments as explanatory factors (comment #16), and about the correctness of the sentiments extracted (comment #17).

#### 7.1.2 Explanations Are Sometimes Not Useful

The topics of vaccination (14 comments) and Catalonia independence (11 comments) have the most comments suggesting that the explanations are not useful to decide on video usefulness. Participants do not find the explanations useful because they *1)* decide based on the video source alone (comment #18), *2)* find the video clear enough (comment #19) or *3)* find the explanations ambiguous (comment #20), superficial (comment #21), or not showing the true stance of the video (comment #22). Category *3)* of comments is often encountered for Supplementary Video V3, which is very often perceived as not useful, satire, or parody.

### 7.2 Analysis of General Comments

In total, we analyzed 157 comments in the condition w/o explanations and 165 comments in the condition w/explanations. Several comments discuss the implications of certain aspects when deciding on video usefulness, such as the belief strength on the topic of the participants, source and speaker trustworthiness, video informativeness, among others. An overview of such themes and comments is given in Table 8.

**TABLE 8:** Example of general comments given by participants at the end of each study condition.

Theme	Comment Id	Comment excerpts (video id)
Polarization	#23	*I would say the video subtitles and comments helped as they seemed a little ambiguous which lead me to believe that although some truth was spoken in the video not all of it was factual …* (CI3)
Reliable & Recent Sources	#24	*know that it was published to a very reputable news channel, like BBC gave it a great deal of credibility* (FT3)
	#25	*I don’t really trust RT, since I believe that is Russia Today, which is operated by the Russian government. I am not 100% sure of their editorial independence, but I’m also not sure what kind of interests they might have in Spain.* (CI3)
	#26	*RT has a pro russian stance on a lot its reports. That doesnt mean this clip should be ignored but if its a discussion to be fair and informed you will want to seek other sources* (CI3)
	#27	*The video seems to be too old to have any relevant information* (FT2)
Objective & Diverse Views	#28	*The interview questions are fair, but the majority of information given is the opinion of one party, who is not neutral or unbiased, so it is not a balanced or comprehensive source for forming an informed opinion on the topic.* (FT1)
	#29	*It presents facts and opinions from both side* (V1)
	#30	*It’s an interview. There are many opinions on both sides of Brexit. The interview let people know what the PM was trying to do.* (FT1)
	#31	*The statistics of trade from different continents vs. Africa was the most convincing for me.* (FT3)
	#32	*The video gave a very good and simple description of how vaccines protect us from disease and what happens when children are not immunized. The illustrations are very good in this video and very useful for understanding of the topic.* (V1)
	#33	*I think the video was really well made because it had infographics in the background, which made it easier for viewers.* (FT3)
Speaker Trustworthiness	#34	*She is reasonable and articulate. She is persuasive in her role as compromiser. I trust her statements.* (FT1)
	#35	*This was just one man speaking and seemed very subjective without being backed by evidence\enleadertwodots* (FT2)
Informativeness	#36	*Sarcasm doesn’t really give facts that people can evaluate rationally* (V3)
	#37	*The video is supposed to be humorous/satire, so while I ultimately I believe the message is good and accurate, but it could be slightly confusing for some, and is not the best, most balanced and straightforward source of information on this topic.* (V3)
	#38	*I couldn’t tell if it was supposed to be satirical or literally inform people. It had neither reputation nor expertise to lend to its credibility. I’d have to see some other stuff from the channel to decide.* (V3)
	#39	*I thought it was interesting but didn’t really give enough information* (CI1)
	#40	*The information in the video may be accurate, but it is primarily from one single source, one person’s argument or opinion which may be biased, so it is likely not the best or most comprehensive source of information on the topic. It does not provide enough background or general information on the topic.* (FT2)

Explanations helped to assess polarized videos. Videos can potentially contain polarizing information that study participants with weaker beliefs on the topic (low values for belief strength on the topic) can only grasp from the explanations we provide regarding user comments, c. f. comment #23. Similarly, the explanations regarding the channel help people decide on the usefulness (comment #24) or uselessness (comments #25 and #26) of the videos. We also saw that reflection triggers indicating recency were useful when the video seems to be dated. Comments such as #27 appear in the condition with explanations, which emphasizes that such explanations trigger participants’ reflection.

Participants’ comments also suggest that not having a strong (extreme) belief strength for a topic influences the perceived video usefulness (comment #28). Then, participants tend to appreciate the objectivity and diversity of viewpoints in a video and thus, align with the video content–comments #29, #30. Statistics and objective facts are also convincing (comments #31-#33). Similarly, participants rely on the speaker trustworthiness to decide on the video usefulness, comment #34 versus #35. Furthermore, participants find a video not useful when the video is confusing, and people can not understanding its true nature (comments #36-#38) and when the video is not informative enough (comments #39, #40).

## 8 Discussion

In this section, we discuss 1) our key results on people’s alignment with the video and belief strength on the topic, explanation usefulness, and reflection in the context of existing cognitive science theories; and 2) the implications for explanations design going forward. We discuss these in light of raising awareness regarding avoiding online deception while preserving end-user privacy. Recall that the videos from our user study were relevant for controversial topics, and they varied in terms of 1) the extent they were expected to evoke emotional responses (stronger emotional responses for vaccination and Catalonia independence, compared to free trade), and 2) how much knowledge people have about them (less expected knowledge on Catalonia independence and free trade, compared to vaccination).

While our goal was to provide explanations for videos as a means of helping people better reflect on the content and thus make better use of it, in practice, we observed that people did not always internalize the information available. Despite the varying emotional or informational motivations of our video topics, human factors (*e.g.*, heuristics and biases) emerged as important considerations. Prior work in cognitive science supports this overarching finding (Simon, 1955). calls this selective internalization of information *bounded rationality*: “broadly stated, the task is to replace the global rationality of economic man with the kind of rational behavior that is compatible with the access to information and the computational capacities that are actually possessed by organisms, including man, in the kinds of environments in which such organisms exist” (Simon, 1955, *p*.99). Our work provides novel insights for how people apply bounded rationality in the online media context despite being provided with explanations. Our findings suggest several reasons on why avoiding online deception is a difficult skill to acquire, but they also provide a practical foundation to raise awareness about online deception. We first discuss these findings below, and then discuss their implications for design of explanations going forward (Section 8.6).

### 8.1 Alignment With the Video and Belief Strength on the Topic

Our quantitative and qualitative analyses suggested that for more ‘emotional’ topics, participants are more prone to demonstrate alignment (agreement) with a video, and where users have a stronger positive belief strength on the topic, alignment is even more likely to occur. Alignment is not likely to occur on topics where participants have less strong beliefs, and likely limited previous knowledge.

Theories from cognitive science could help us understand this result in the online media context, if we see it as an example of how previous knowledge can influence how content is perceived. Given a situation where people’s attitudes, beliefs, or behaviors are questioned by providing additional information, people tend to justify or rationalize their perspective by only paying attention to the information that supports it. This behavior is characterized as the use of the availability heuristic, *i.e.*, people’s tendency to use information that comes to mind quickly and easily when making decisions about the future (Tversky and Kahneman, 1973). The application of the availability heuristic also exacerbates confirmation bias in how people perceive these online videos: people interpret new information (*e.g.*, in the form of explanations for topics on which they feel strongly - belief strength) as confirmation of one’s existing beliefs or theories, despite the content of the information (Nickerson, 1998). We hypothesize that this use of the availability heuristic, which leads to confirmation bias, is critical to how people perceive explanations about videos—our results show some initial evidence that this might be the case. We include specific design implications based on this finding in Section 8.6.

### 8.2 Useful Reflection Triggers

We found that the source of the videos is the trigger most frequently mentioned as being informative to decide on video usefulness. The trigger regarding the sentiment and emotions evoked by the video comments, in comparison with the video subtitles, is also helpful when participants do not have a lot of knowledge on the topic. Such a trigger helps them understand that the video is biased, or exposes a single viewpoint. Specifically for topics on which participants have moderate belief strength (less assumed knowledge), explanations help people to decide that the video is useful when the topic is also less emotional (free trade), and not useful when the topic is more emotional (Catalonia independence). While in the user study we selected videos for which such triggers could be generated, our dataset of 960 videos contains 283 videos without user comments and 576 videos that have no topic overlapping between video subtitles and video comments. Thus, when we cannot generate reflection triggers referring to emotions and sentiments, the explanations could be evaluated as not containing enough information. Nevertheless, the reflection triggers we selected span a large range of attributes, and in the absence of certain reflection triggers, the available ones can be inspected.

### 8.3 Explanations Quality

Explanations were found to be useful overall and of high quality (see Table 6). However, they seem to show more information than needed, which also emerged from the qualitative analysis of the comments. Very few participants mention more than one reflection trigger as being helpful to decide on the usefulness of a video. The source of the video, *i.e.*, the first-mentioned reflection trigger, is found useful the most. Furthermore, explanations are less useful and qualitative when they do not capture the true nature of the video (see the sarcastic Supplementary Video V3 and user comments #36-#38 about it).

Prior work in cognitive science highlights people’s propensity to anchor to specific pieces of information rather than internalizing all information. Kahneman and Tversky classify this behavior as the use of the *anchoring heuristic*: the tendency to accept and rely on the first piece of information received before making a decision. That first piece of information serves as the anchor and sets the tone for any decisions that follow (Tversky and Kahneman, 1974). As such, we anticipate different types of information content to serve as the anchor, depending on the individual. While in our user study we saw the source of the video to be the most common anchor, a larger-scale evaluation in future work might highlight other patterns of behavior. Similar to the availability heuristic, the anchoring heuristic also often results in confirmation bias in decision-making contexts. We describe some implications for design resulting from this in Section 8.6.

### 8.4 Reflection

The qualitative analysis showed that reflection indeed emerges when study participants see the explanations, especially on topics where they have less knowledge. Participants analyze the differences in sentiment and emotion perception between video subtitles and video comments. Large differences between the two make participants believe that the video could be biased towards showing a limited number of viewpoints, which hinders the perceived usefulness of the video. Explanations regarding the source and the publishing date of the video make people reflect on the relevance and recency of the information presented in the video, *i.e.*, older videos or issues can be outdated.

It is important to note the difference between these results and the ones above that describe the situations where people do have prior knowledge and beliefs about a topic, and specific alignment with the video. While those provide evidence for the application of some common heuristics and biases in the online media context, these results for people who are not as knowledgeable or opinionated about the topic are supported by what we know of people’s overarching sensemaking process. Sensemaking is most prominent in discrepant events, or surprises that trigger a need for explanation. People try to apply their existing cognitive frameworks particularly when predictions or expectations break down (Weick, 1995). Indeed, this is what we see in the case with limited belief strength described above.

### 8.5 Privacy-Preserving Approach

The design of our approach is privacy-preserving for the two key stakeholders involved, namely the users that consume the information provided by the explanations (study participants) and the users that provided comments on the videos we analyze. Regarding the former, we do not ask for study participants’ stance on the video topic to try and persuade them into changing their opinion. Similarly, for the users that provide comments, we do not include their information in the explanations—we do not provide samples of comments and their analysis in terms of communicated emotions or sentiment. Instead, our explanations foster reflection through a neutral mean of presenting additional information regarding the video and by providing the aggregated opinion of all users that watched the video and provided comments.

### 8.6 Design Implications

Our study suggests several improvements to the generated explanations. They also help us better understand when and how these explanations contribute to reflective assessment.

#### 8.6.1 When Are Explanations Useful?

The user study showed that participants with moderate belief strength (and likely limited knowledge on a topic) are more prone to perceive a video as useful if the video provides rich and diverse viewpoints. These participants used the reflection triggers describing the emotions and the sentiments evoked by the video subtitles and video comments to reflect on the content of the video (*i.e.*, to understand how topics are depicted in the video and how they are perceived by users). However, both participants with moderate and extreme belief strength appreciate the reliability of the aspects discussed in the video. Explanations also seem to specifically influence reflective assessments of videos when people have strong beliefs on a topic but are not as effective as one might hope. Nevertheless, *explanations helped our participants to identify polarizing videos, which is a key aspect in avoiding online deception*.

#### 8.6.2 How to use Explanations?

Our results indicate that the alignment with the video was more important for evaluating video usefulness than the *belief strength* on the topic, or the content of the *explanations*. *This would suggest that people’s preconceived notions about a topic and whether a video supports vs. opposes these are critical to how they evaluate video usefulness.* Thus helping users who have preconceived, strong beliefs on a topic with avoiding online video deception is even more challenging. Further investigation into this aspect is needed. One solution would be to better explore the role of explanations on building user trust, helping them to make better decisions (*i.e*, evaluate the effectiveness of the explanations), and persuading them on further reflection on the information. These aspects regarding explanations could be adapted from well-studied work on recommender systems (Tintarev and Masthoff, 2012).

These results are supported by prior work on cognitive dissonance: a feeling of mental discomfort and psychological stress experienced in situations where people are introduced to conflicting attitudes, beliefs, or behaviors compared to their own (Festinger, 1957). In our case, we observed that people with an extreme negative alignment with a video found it to be less useful, with or without the explanation. Indeed, attitude polarization of this kind is a common byproduct of cognitive dissonance (Tesser and Conlee, 1975; Bail et al., 2018). In line with our observations about *negative* alignment, prior work in controversial domains (*e.g.*, politics) also suggests that this attitude polarization is asymmetric, with negative alignment being far more polarizing (Hacker and Pierson, 2015).

Given our results, a key design implication for explanations in future work is the need for tailoring them to people’s alignment with a video, rather than their topic belief strength or the video content itself. Simply providing information (as explanations) is only one aspect—how the information is presented is critical to whether people internalize it or reflect on it (Mulder et al., 2021; Rieger et al., 2020). Other ways in which people resolve cognitive dissonance include diverting their attention away from their dissonant conditions, trivializing the dissonant information and self-affirmation, denying responsibility of understanding the information, and, on the rare occasion, changing their attitude and behavior (Brehm and Cohen, 1962; McGrath, 2017). Future work must consider this range of potential behaviors when designing explanations.

In practice, our proposed explanations and reflection triggers could be used along-side videos, *e.g.*, on YouTube. We could use personalized user information (*e.g.*, videos watched, videos and topics of interest, opinion on videos and topics) to address the aforementioned design implications. Such user models, however, should be designed in-line with privacy-preserving degrees expected by users. In addition to fostering self-reflection, proposed explanations could serve the purpose of developing users’ information literacy (Hahnel et al., 2020), to inform or make users aware of the potential extreme or unscientific viewpoints expressed in the recommended YouTube videos (Spinelli and Crovella, 2020). Inspired by video summarization approaches (Chen et al., 2017), we argue that parts of our reflection triggers could be linked to particular moments in the video. We could inform viewers of opposing emotions regarding the perception of a topic at a particular moment in the video and in video comments. While video summaries offer quick and concise video overviews, they could potentially miss relevant and important information and thus lead to misinformation and deception. Graphical explanations can support users in better understanding how representative video summaries are for the original video (Inel et al., 2020). With our reflection triggers, consumers could also actively reflect on the content of the videos, while watching a summary.

#### 8.6.3 Which Reflection Triggers Are Useful?

The source of the video was found useful by the majority of the participants. However, only participants with limited knowledge on a topic found the sentiment and emotions triggers relevant. This suggests, as in Section 8.6.2, that explanations and reflection triggers need to be better tailored for different types of users, but also for various types of videos—see the example of the sarcastic V3 video. Thus, future studies should focus on determining a set of relevant reflection triggers for various purposes, such as accounting for users’ prior knowledge and beliefs, video types, the purpose of using the video, among other.

## 9 Limitations

We identify several limitations in this work, regarding *1)* the pipeline for generating reflective explanations, *2)* the choice of reflection triggers for explanations and *3)* the experimental setup.

We note that while this experiment required that we take specific decisions regarding the experimental design (*e.g.*, topics or videos to study, subset of possible reflection triggers), the presented pipeline is open source and readily extendable for a wide range of experiments to mitigate deception in online videos.

### 9.1 Pipeline

The explanation generation pipeline is fully automated, by connecting several off-the-shelf tools, such as Google Video Intelligence and Watson NLU. While the pipeline is easy to replicate and extend, we acknowledge that issues of one component could affect the accuracy of another component (*e.g.*, wrong speech-to-text transcription affects the identification of key entities). Furthermore, speech-to-text tools may have lower accuracy for people with accents or non-native English speakers, while entity recognition tools may not identify entities that are seen for the first time. Similarly, both sentiment and emotion are culture-specific aspects, and different pieces of information could evoke different sentiment and emotion. We try to minimize these issues by *1)* focusing on known topics and issues, that contain entities likely to be recognized by information extraction tools and *2)* presenting aggregate views of sentiment and emotion. Our empirical analysis and literature review also showed that the tools perform well on our tasks. Even though we applied our methodology on a set of 960 videos, the pipeline is video-agnostic. Our GitHub repository provides details on how to replicate our approach on a set of videos. Since storing transcriptions is not compliant with API ToS, we provide guidelines on how to retrieve them. NB: such transcriptions and output from other APIs could be slightly different now, as the APIs are updated and improved.

We also simplified our approach by looking into the collective sentiment and emotion of all video comments. However, we could also study variations in a person’s emotions and see which topics might have triggered changes in a person’s behaviour or emotion. Currently, we consider equally relevant all entities mentioned in both video subtitles and video comments. Future research could focus on ranking these key entities based on their relevance to the video, as suggested by our qualitative analysis. Furthermore, while entities such as people, locations, and organizations, can contextualize well information in videos (Gligorov et al., 2011), we agree that our video understanding could be improved. Future work could focus on identifying more topic-specific concepts, facts or statements, instead of these very granular entities.

### 9.2 Reflective Triggers and Explanations

The literature provides a range of reflection triggers that we could apply. We yet only focus on channel reputation, key entity, sentiments and emotions, and controversiality. Furthermore, we understood that participants prefer also explanations that describe the true nature of a video, to help them differentiate between different video types (documentaries, news clips, satire, among others) and better assess the usefulness of videos. In Section 4.5, we acknowledge that videos may not have comments, in which case we cannot generate explanations regarding the emotions and sentiments expressed in the video comments. Therefore, in such cases, users might not be able to properly reflect on the emotional controversiality of the video and ultimately, on the usefulness of the video. Furthermore, we acknowledge that user comments are not representative for all people that watch a video and people with strong opinions are more likely to comment. It is also likely that users provide irrelevant information in their comments (Shetty et al., 2020; Kavitha et al., 2020). We try to alleviate these issues by only analyzing comments that have entities in common with the video, and are not comment replies. Nevertheless, comments provide insights into the video watching experience of users, in a way that seems unattainable otherwise.

In addition, for each reflection trigger, we only extract one type of information and generate one type of explanation. Regarding emotions, we could also provide a list of extremely polarized comments, or ranges of emotion values across users. However, we argue that these aggregated views are more suitable to promote awareness regarding users’ opinion on the video, while preserving their privacy. We also do not consider comments’ temporality, which means that we can not observe how emotion and sentiment towards certain entities change over time. As such, the overview of sentiment and emotions that we present are representative for a certain snapshot in time—these values are likely to change with new comments.

### 9.3 Setup

To reduce the evaluation load of crowd participants, we limited the maximal duration of the videos. Short length videos, however, give us the necessary confidence that study participants could focus on the entire video duration. Although we only used three controversial topics and three videos per topic, we can apply our pipeline for generating natural language explanations for reflective assessment to any controversial topic and video. Furthermore, the three topics that we chose to vary in terms of the amount of knowledge people have on them, and in terms of the emotional impact that they can have on people, which apply to many topics. Future work must consider applying and evaluating our pipeline for videos on different topics, as well as longer viewing sessions.

In our study, we did not control for participants’ opinions on the topic. We allowed both people that have strong and weak opinions on the topic to participate. We believe this is a natural condition for our scenario, which asks participants to imagine that they need to research a topic on YouTube and then use it in a discussion. We account for participants’ prior knowledge indirectly, by asking them how strong they feel about a topic, instead of asking whether they have any background knowledge on the topic. However, we consider it is acceptable to believe that for someone to have an opinion on a topic, s(he) needs at least some prior knowledge. We agree that it would be clearer to disambiguate between having some knowledge and being knowledgeable.

For each video, we described the same reflection triggers, which allowed for a balanced design across videos. However, this also meant that for some videos, the same reflection trigger conveyed positive information, and for others, negative information. The current experimental design did not allow us to study the polarity of individual reflection triggers or even the weighting of the reflection triggers.

## 10 Conclusion and Future Work

In this paper, we introduced an end-to-end approach for automatically generating natural language explanations to foster reflective assessment of online videos on controversial topics. We can replicate the pipeline for any controversial topic, and videos of any length. We distinguish from work dealing with credibility assessment of online information by *1)* focusing on less researched sources such as videos, and by *2)* helping people to reason about the videos they watch, instead of providing a credibility measure or score. We do not aim to provide a clear answer concerning the video content or to push a particular evaluation of content, but we present a neutral means for participants to judge the video usefulness by themselves and raise their awareness regarding potential deceiving information the video contains.

We evaluate the impact of these explanations in a between-subjects user study with two conditions, video without and with explanations. Participants found the explanations useful to very useful. This result was weaker, but still positive, for topics on which they did not have a strong belief. While a strong belief (and possibly previous knowledge) about the topic did not influence perceived video usefulness, viewer alignment with the video did. The increase in perceived video usefulness was not significant in the condition with explanations, but the explanations helped participants to make an informed decision on topics on which they have limited belief and likely limited knowledge. We also evaluated the generated explanations regarding their effectiveness to communicate the intended information, using Gricean Maxims. Participants found the explanations to contain enough information, provide truthful information, be relevant, clear, and without ambiguity. We also found that each reflection trigger is relevant when deciding on video usefulness, with the source of the video being the most frequently mentioned, followed by the sentiment and emotions evoked in the video subtitles and video comments.

These results suggest that the generated explanations are a good foundation for supporting reflective assessment. In future work, we will analyze a more extensive set of key entity types and include topic-specific types, with an in-depth analysis of their relevance, as well as to perform a more in-depth analysis of the comments, such as in terms of temporality. In our user study, we have also identified potential candidates for additional reflection triggers. For example, participants showed interest in understanding the objectivity and the diversity of viewpoints, as well as the trustworthiness of the speakers. Extracting such aspects from the video could help participants with limited knowledge on a topic. Participants also suggest that the explanations should capture the true nature of the video and inform them about the video type (*e.g.*, credible or non-credible video, factual or satirical video). Thus, we plan to experiment with different types of videos, *i.e.*, documentaries, news, satire, to understand what kind of reflection triggers and explanations are suitable for different kinds of videos, to foster viewers reflection. Finally, we would like to experiment with various styles for presenting the explanations, tailoring them to level of alignment and individual characteristics of users (*e.g.*, accuracy motive).

## Data Availability

The datasets, notebooks, user studies, and analyses presented in this paper can be found at the following repository: https://github.com/oana-inel/Explanations-ReflectionTriggers-Videos and in the article Supplementary Material.

## Footnotes

1
https://blog.youtube/news-and-events/youtube-at-15-my-personal-journey.

2 The data, notebooks and analyses performed for this research are publicly available at https://github.com/oana-inel/Explanations-ReflectionTriggers-Videos.

3
https://chrome.google.com/webstore/detail/fake-video-news-debunker/mhccpoafgdgbhnjfhkcmgknndkeenfhe?hl=en, retrieved September 2019.

4
http://en.wikipedia.org/wiki/Wikipedia:List_of_controversial_issues, retrieved May 2019.

5
https://developers.google.com/youtube/v3/docs/, retrieved June 2019.

6
https://cloud.google.com/video-intelligence/docs/transcription, retrieved May 2019.

7
https://cloud.ibm.com/apidocs/natural-language-understanding, version from 2018 to 11-16, June 2019.

8
https://www.kdnuggets.com/2018/08/comparison-most-useful-text-processing-apis.html, retrieved December 2019.

9
https://cloud.ibm.com/docs/natural-language-understanding?topic=natural-language-understanding-entity-types-version-1, retrieved September 2019.

10
https://www.nltk.org/book/ch05.html, retrieved September 2019.

11
https://www.nltk.org/_modules/nltk/stem/wordnet.html, retrieved September 2019.

12
http://en.wikipedia.org/wiki/Wikipedia:List_of_controversial_issues, retrieved September 2019.

13
https://pypi.org/project/SPARQLWrapper/, retrieved September 2019.

14 The user study was approved by the ethics committee before publication.

15
https://www.mturk.com.

16 Nor for any topic. Mann-Whitney U tests: vaccination–t = 5,350.5, *p* > 0.05, Catalonia independence–t = 5,328.5, *p* > 0.05, free trade–t = 5,017.0, *p* > 0.05).

17 Note here that some of the participants used the two comment fields interchangeable, so we tried to be inclusive when identifying themes.

18 We note here that we marked all comments “None” as “not useful”.
