# Supplementary material for: Design Implications for Explanations: A Case Study on Supporting Reflective Assessment of Potentially Misleading Videos
Source: Front Artif Intell. 2021 Sep 27;4:712072. doi: 10.3389/frai.2021.712072 (PMC8507585; doi:10.3389/frai.2021.712072)
Supplement: Supplementary file 1 [file DataSheet1.PDF]

frontiers

Supplementary Material

1 SUPPLEMENTARY DATA

The supplementary data contains examples of generated explanations, for the nine videos used in the user study.

1.1 Explanation for Video V1

- The *channel* where the video is published has **6,487,834 subscribers**.
- The *channel* where the video is published was created on **2014-03-04**. The *video* above was posted on **2015-02-12**.
- The *channel* where the video is published is **related to the following channel(s): Ezra Klein Show, Recode, The Verge, Verge Science, Eater, SB Nation, Curbed, Polygon**.

- The *video* is related to the following *topic(s)*: **polio, us, smallpox, Brooklyn, Edward Jenner, Ohio, Rand Paul, Philippines**.
- The following *2 topic(s)* mentioned in the video was/were considered *controversial on Wikipedia*: **us, Philippines**.

- The *comments of the video* express the following *emotion(s)*: **anger, sadness, fear, disgust**.
- The *comments of the video* express the following *emotion(s)* towards the *topic(s)* mentioned above:
  - **anger: us, Edward Jenner**
  - **disgust: us, Brooklyn, Rand Paul**
  - **fear: polio, us, smallpox, Brooklyn, Philippines**
  - **joy: polio, smallpox, Edward Jenner, Ohio**
  - **sadness: polio, us, smallpox, Rand Paul, Philippines**

- The *video subtitles and the video comments* express the following *sentiment(s)* towards the *topic(s)* mentioned above:
  - **Brooklyn, Rand Paul**: depicted as **neutral** in the *video subtitles* and as **negative** in the *video comments*.
  - **polio, us, smallpox, Edward Jenner, Ohio, Philippines**: depicted as **negative** in the *video subtitles* and as **negative** in the *video comments*.

1.2 Explanation for Video V2

- The *channel* where the video is published has **2,410,215 subscribers**.
- The *channel* where the video is published was created on **2014-07-02**. The *video* above was posted on **2019-02-24**.
- The *channel* where the video is published is **related to the following channel(s): grande1899, More Grandayy, grandayy malta, Dolan Dark, FlyingKitty, Gus Johnson, SethEverman, VoiceoverPete, PewDiePie, HowToBasic**.

- The *video* is related to the following *topic(s)*: **polio, Andrew Wakefield, Karen, Dr Phil**.
- The following *1 topic(s)* mentioned in the video was/were considered *controversial on Wikipedia*: **Dr Phil**.

- The *comments of the video* express the following *emotion(s)*: **joy**.
- The *comments of the video* express the following *emotion(s)* towards the *topic(s)* mentioned above:
  - **anger: Andrew Wakefield**
  - **disgust: Andrew Wakefield**
  - **fear: polio**
  - **joy: polio, Andrew Wakefield, Dr Phil**
  - **sadness: polio, Andrew Wakefield**

- The *video subtitles and the video comments* express the following *sentiment(s)* towards the *topic(s)* mentioned above:
  - **Dr Phil**: depicted as **negative** in the *video subtitles* and as **positive** in the *video comments*.
  - **polio, Andrew Wakefield, Karen**: depicted as **negative** in the *video subtitles* and as **negative** in the *video comments*.

1.3 Explanation for Video V3

- The *channel* where the video is published has **439,000 subscribers**.
- The *channel* where the video is published was created on **2011-08-17**. The *video* above was posted on **2016-03-11**.
- The *channel* where the video is published is **related to the following channel(s): SourceFed, SourceFedNERD, BREEessrig, Matt Lieberman, Steve Zaragoza**.

- The *video* is related to the following *topic(s)*: **America, Autism, England, Doug**.
- The following **2** *topic(s)* mentioned in the video was/were considered *controversial on Wikipedia*: **Autism, England**.

- The *comments of the video* express the following *emotion(s)*: **anger, joy**.
- The *comments of the video* express the following *emotion(s)* towards the *topic(s)* mentioned above:
  - **anger: Autism**
  - **disgust: America, England**
  - **fear: America, Autism**
  - **joy: America, England, Doug**
  - **sadness: America, Autism**

- The *video subtitles and the video comments* express the following *sentiment(s)* towards the *topic(s)* mentioned above:
  - **England**: depicted as **positive** in the *video subtitles* and as **neutral** in the *video comments*.
  - **Autism**: depicted as **negative** in the *video subtitles* and as **neutral** in the *video comments*.
  - **America, Doug**: depicted as **negative** in the *video subtitles* and as **negative** in the *video comments*.

1.4 Explanation for Video Cl1

- The *channel* where the video is published has **7,510,000 subscribers**.
- The *channel* where the video is published was created on **2005-10-02**. The *video* above was posted on **2017-10-01**.

- The *channel* where the video is published is **related to the following channel(s): CNN Politics, CNNLivestreams, The Daily Share, Adult Swim, TBS, TNT, Team Coco, Cartoon Network**.
- The *video* is related to the following *topic(s)*: **Catalonia, Madrid**.
- The following *1 topic(s)* mentioned in the video was/were considered *controversial on Wikipedia*: **Catalonia**.
- The *comments of the video* express the following *emotion(s)*: **anger, fear, disgust**.
- The *comments of the video* express the following *emotion(s)* towards the *topic(s)* mentioned above:
  - **anger: Catalonia, Madrid**
  - **disgust: Catalonia, Madrid**
  - **fear: Catalonia**
  - **joy: Catalonia**
- The *video subtitles and the video comments* express the following *sentiment(s)* towards the *topic(s)* mentioned above:
  - **Catalonia**: depicted as **negative** in the *video subtitles* and as **positive** in the *video comments*.
  - **Madrid**: depicted as **negative** in the *video subtitles* and as **negative** in the *video comments*.

1.5 Explanation for Video CI2

- The *channel* where the video is published has **5,041,096 subscribers**.
- The *channel* where the video is published was created on **2006-04-08**. The *video* above was posted on **2017-09-22**.
- The *channel* where the video is published is **related to the following channel(s): BBC Reel, BBC Click, BBC HARDtalk, BBC Newsnight, BBC, BBC Newsbeat, BBC Studios, BBC Earth, BBC News Mundo, BBC News Brasil, BBC Persian, BBC News Hindi, BBC Pashto, BBC Stories, BBC Ideas**[1].
- The *video* is related to the following *topic(s)*: **Catalonia, Spain, Barcelona**.
- The following *2 topic(s)* mentioned in the video was/were considered *controversial on Wikipedia*: **Catalonia, Spain**.
- The *comments of the video* express the following *emotion(s)*: **joy**.
- The *comments of the video* express the following *emotion(s)* towards the *topic(s)* mentioned above:
  - **anger: Catalonia**
  - **disgust: Spain**
  - **fear: Catalonia**
  - **joy: Catalonia, Spain, Barcelona**
  - **sadness: Catalonia, Spain**
- The *video subtitles and the video comments* express the following *sentiment(s)* towards the *topic(s)* mentioned above:
  - **Barcelona**: depicted as **positive** in the *video subtitles* and as **positive** in the *video comments*.
  - **Spain**: depicted as **positive** in the *video subtitles* and as **negative** in the *video comments*.

[1] Some related channels were omitted from the list due to unrecognized characters.

- **Catalonia**: depicted as **negative** in the *video subtitles* and as **negative** in the *video comments*.

1.6 Explanation for Video CI3

- The *channel* where the video is published has **3,670,000 subscribers**.
- The *channel* where the video is published was created on **2007-03-28**. The *video* above was posted on **2013-09-12**.
- The *channel* where the video is published is **related to the following channel(s): RT Sport, In the NOW, RT America, RT Documentary, Watching the Hawks RT, Redacted Tonight, WorldsApaRT, RT UK, Ruptly, goingundergroundRT, RT Chinese, RT Arabic, RT France, RT Deutsch, Boom Bust, The Big Picture RT, breakingtheset, LearnRussianwithRT, primetimeru, Raw Take**[2].
- The *video* is related to the following *topic(s)*: **Catalonia, Spain, Barcelona, Europe**.
- The following *3 topic(s)* mentioned in the video was/were considered *controversial on Wikipedia*: **Catalonia, Spain, Europe**.
- The *comments of the video* express the following *emotion(s)*: **anger, sadness, fear**.
- The *comments of the video* express the following *emotion(s)* towards the *topic(s)* mentioned above:
  - **disgust: Europe**
  - **fear: Europe**
  - **joy: Catalonia, Barcelona, Europe**
  - **sadness: Europe**
- The *video subtitles and the video comments* express the following *sentiment(s)* towards the *topic(s)* mentioned above:
  - **Catalonia**: depicted as **neutral** in the *video subtitles* and as **positive** in the *video comments*.
  - **Spain, Europe**: depicted as **neutral** in the *video subtitles* and as **negative** in the *video comments*.
  - **Barcelona**: depicted as **negative** in the *video subtitles* and as **positive** in the *video comments*.

1.7 Explanation for Video FT1

- The *channel* where the video is published has **541,797 subscribers**.
- The *channel* where the video is published was created on **2012-08-20**. The *video* above was posted on **2018-07-06**.
- The *channel* where the video is published is **related to the following channel(s): DailyMailTV, Daily Mail Sport**.
- The *video* is related to the following *topic(s)*: **European Union, UK, Parliament**.
- The following *2 topic(s)* mentioned in the video was/were considered *controversial on Wikipedia*: **European Union, UK**.
- The *comments of the video* express the following *emotion(s)*: **anger, fear, disgust**.
- The *comments of the video* express the following *emotion(s)* towards the *topic(s)* mentioned above:
  - **anger: UK**
  - **disgust: UK**

[2] Some related channels were omitted from the list due to unrecognized characters.

- **fear: UK**
- **joy: European Union**
- **sadness: European Union, UK, Parliament**

- The *video subtitles and the video comments* express the following *sentiment(s)* towards the *topic(s)* mentioned above:
  - **European Union**: depicted as **positive** in the *video subtitles* and as **positive** in the *video comments*.
  - **UK, Parliament**: depicted as **positive** in the *video subtitles* and as **negative** in the *video comments*.

1.8 Explanation for Video FT2

- The *channel* where the video is published has **31,173 subscribers**.
- The *channel* where the video is published was created on **2007-01-22**. The *video* above was posted on **2019-02-12**.
- The *channel* where the video is published is **related to the following channel(s): Nigel Farage**.

- The *video* is related to the following *topic(s)*: **UK, EU, Germany, Singapore**.
- The following *3 topic(s)* mentioned in the video was/were considered *controversial on Wikipedia*: **UK, EU, Germany**.

- The *comments of the video* express the following *emotion(s)*: **anger, sadness, fear, disgust**.
- The *comments of the video* express the following *emotion(s)* towards the *topic(s)* mentioned above:
  - **anger: UK, EU, Germany**
  - **disgust: EU, Germany**
  - **fear: UK, EU**
  - **joy: Singapore**
  - **sadness: UK, EU, Germany, Singapore**

- The *video subtitles and the video comments* express the following *sentiment(s)* towards the *topic(s)* mentioned above:
  - **Germany**: depicted as **neutral** in the *video subtitles* and as **negative** in the *video comments*.
  - **UK, EU, Singapore**: depicted as **positive** in the *video subtitles* and as **negative** in the *video comments*.

1.9 Explanation for Video FT3

- The *channel* where the video is published has **5,041,096 subscribers**.
- The *channel* where the video is published was created on **2006-04-08**. The *video* above was posted on **2018-03-21**.
- The *channel* where the video is published is **related to the following channel(s): BBC Reel, BBC Click, BBC HARDtalk, BBC Newsnight, BBC, BBC Newsbeat, BBC Studios, BBC Earth, BBC News Mundo, BBC News Brasil, BBC Persian, BBC News Hindi, BBC Pashto, BBC Stories, BBC Ideas**[3].

- The *video* is related to the following *topic(s)*: **Africa, Europe, European Union, Nigeria, Asia, Kenya**.

[3] Some related channels were omitted from the list due to unrecognized characters.

- The following *4 topic(s)* mentioned in the video was/were considered *controversial on Wikipedia*: **Africa, Europe, European Union, Asia**.
- The *comments of the video* express the following *emotion(s)*: **joy, sadness**.
- The *comments of the video* express the following *emotion(s)* towards the *topic(s)* mentioned above:
  - **anger: European Union, Nigeria**
  - **disgust: Africa, Europe, European Union, Nigeria**
  - **fear: Africa, Europe, Nigeria, Asia**
  - **joy: Nigeria, Asia**
  - **sadness: Africa, Europe, European Union**
- The *video subtitles and the video comments* express the following *sentiment(s)* towards the *topic(s)* mentioned above:
  - **Kenya**: depicted as **neutral** in the *video subtitles* and as **neutral** in the *video comments*.
  - **Asia**: depicted as **neutral** in the *video subtitles* and as **positive** in the *video comments*.
  - **Europe, European Union**: depicted as **neutral** in the *video subtitles* and as **negative** in the *video comments*.
  - **Africa, Nigeria**: depicted as **positive** in the *video subtitles* and as **negative** in the *video comments*.

2 SUPPLEMENTARY FIGURES

In this section of the supplementary material, we provide screenshots of the user study, in the two conditions, without explanations in Figure S1 and with explanations in Figure S2.

2.1 Figures

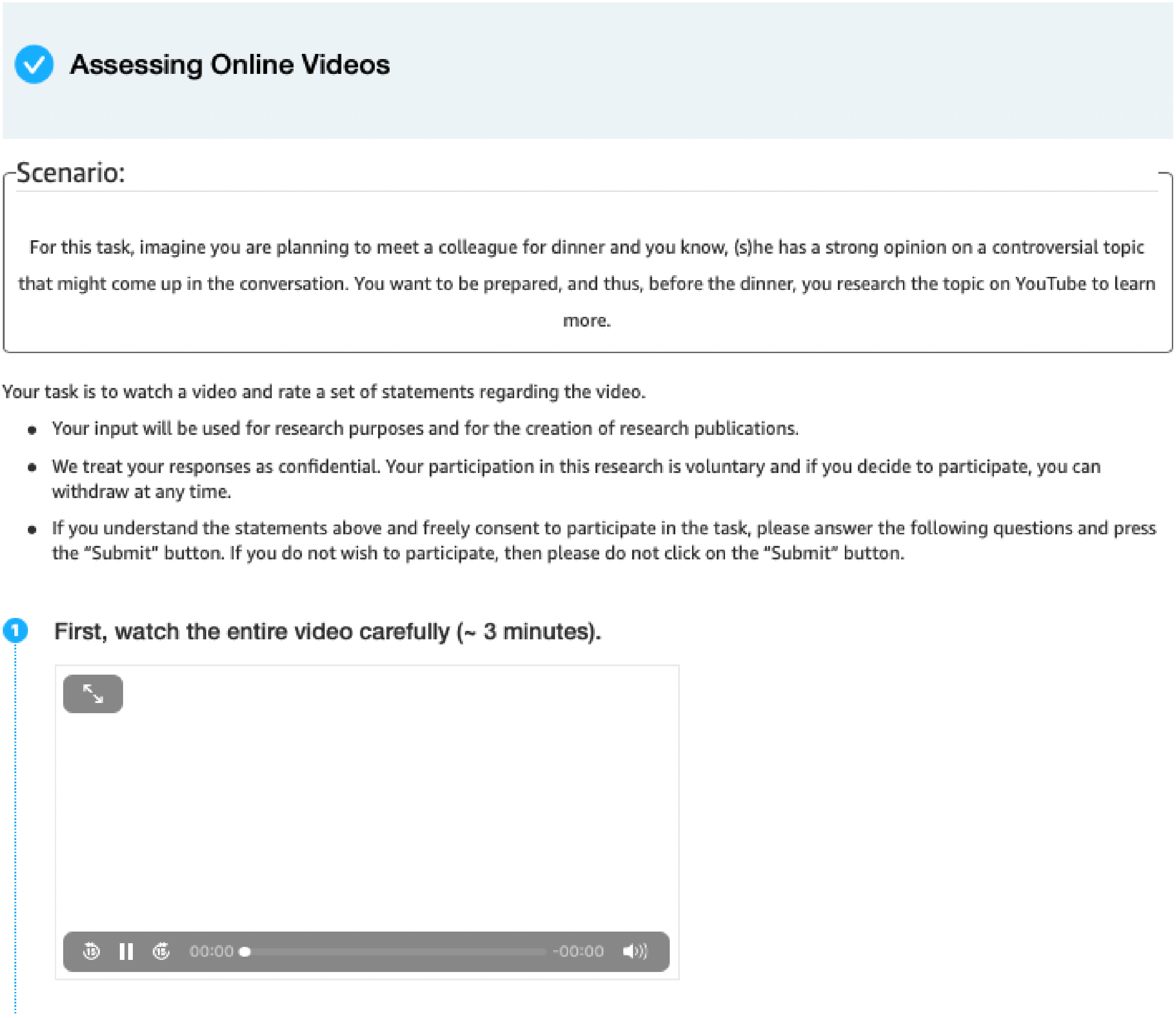

Figure S1. Screenshot of User Study in the Condition without Explanations.

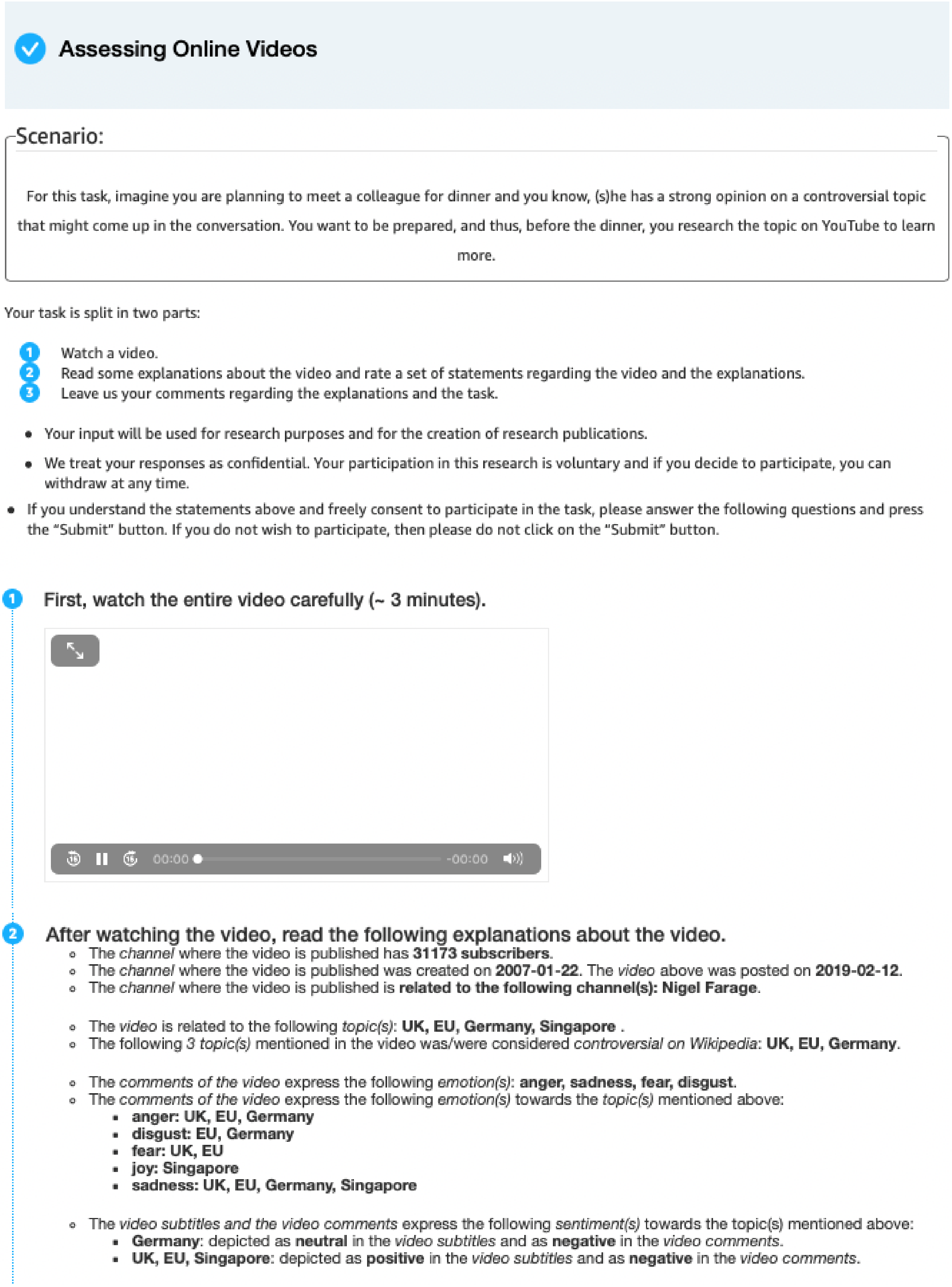

Assessing Online Videos

Scenario:

For this task, imagine you are planning to meet a colleague for dinner and you know, (s)he has a strong opinion on a controversial topic that might come up in the conversation. You want to be prepared, and thus, before the dinner, you research the topic on YouTube to learn more.

Your task is split in two parts:

1. Watch a video.
2. Read some explanations about the video and rate a set of statements regarding the video and the explanations.
3. Leave us your comments regarding the explanations and the task.

- Your input will be used for research purposes and for the creation of research publications.
- We treat your responses as confidential. Your participation in this research is voluntary and if you decide to participate, you can withdraw at any time.
- If you understand the statements above and freely consent to participate in the task, please answer the following questions and press the "Submit" button. If you do not wish to participate, then please do not click on the "Submit" button.

1 First, watch the entire video carefully (~ 3 minutes).

00:00 -00:00

2 After watching the video, read the following explanations about the video.

- The *channel* where the video is published has **31173 subscribers**.
- The *channel* where the video is published was created on **2007-01-22**. The *video* above was posted on **2019-02-12**.
- The *channel* where the video is published is **related to the following channel(s): Nigel Farage**.

- The *video* is related to the following *topic(s)*: **UK, EU, Germany, Singapore** .
- The following *3 topic(s)* mentioned in the video was/were considered *controversial on Wikipedia*: **UK, EU, Germany**.

- The *comments of the video* express the following *emotion(s)*: **anger, sadness, fear, disgust**.
- The *comments of the video* express the following *emotion(s)* towards the *topic(s)* mentioned above:
  - **anger: UK, EU, Germany**
  - **disgust: EU, Germany**
  - **fear: UK, EU**
  - **joy: Singapore**
  - **sadness: UK, EU, Germany, Singapore**

- The *video subtitles and the video comments* express the following *sentiment(s)* towards the topic(s) mentioned above:
  - **Germany**: depicted as **neutral** in the *video subtitles* and as **negative** in the *video comments*.
  - **UK, EU, Singapore**: depicted as **positive** in the *video subtitles* and as **negative** in the *video comments*.

Figure S2. Screenshot of User Study in the Condition with Explanations.
